# N-acetyl-L-leucine normalizes Transcription Factor EB activity by stereospecific bidirectional modulation in a HeLa cell model of Niemann-Pick disease type C

**DOI:** 10.1371/journal.pone.0353834

**Published:** 2026-07-17

**Authors:** Lianne C. Davis, Wim Annaert, Rebecca Braine, Grant C. Churchill, Mallory Factor, Taylor Fields, Marc Patterson, Frances Platt, Dawn Shepherd, Michael Strupp, Antony Galione

**Affiliations:** 1 Department of Pharmacology, University of Oxford, Oxford, United Kingdom; 2 VIB Center for Neuroscience Leuven & Department of Neurosciences, Laboratory for Membrane Trafficking, Leuven, Belgium; 3 IntraBio Inc, Austin, United States of America; 4 Department of Neurology, LMU University Hospital, LMU Munich, Munich, Germany; University College London, UNITED KINGDOM OF GREAT BRITAIN AND NORTHERN IRELAND

## Abstract

Levacetylleucine (Aqneursa™), an acetylated derivative and pro-drug of L-leucine, is the only FDA-approved monotherapy for Niemann–Pick disease type C (NPC). Its acetyl group enables transport via monocarboxylate transporters, supporting blood–brain barrier penetration and efficient cellular uptake. Inside cells, levacetylleucine is metabolised by acylases, generating elevated levels of L-leucine that enhance mitochondrial bioenergetics and is thought to ameliorate lysosomal dysfunction indirectly. Here, we describe a direct effect of levacetylleucine on lysosomal regulation through modulation of TFEB, the master transcription factor for lysosomal and autophagy genes. Levacetylleucine rapidly alters TFEB translocation between the cytoplasm and the nucleus in a biphasic, homeostasis-restoring manner. In wild-type HeLa cells, levacetylleucine promotes TFEB activation and nuclear localisation. However, in NPC1 disease models, where we show that TFEB is over-activated and enriched in the nucleus due to lysosomal stress, levacetylleucine reduces nuclear TFEB and restores a more normal cytoplasmic-to-nuclear balance. These effects occur at clinically relevant concentrations associated with lysosomal storage reduction. The effects of the drug are stereospecific: while the L-enantiomer is active, the D-enantiomer and racemate show no effect, revealing the antagonistic properties of the D-enantiomer. This bidirectional normalisation of TFEB activity highlights a direct mechanism through which levacetylleucine modulates lysosomal and autophagic pathways in the HeLa cell model, giving mechanistic insight into its therapeutic potential in NPC, and also across diverse neurological and neurodevelopmental disorders.

## Introduction

Levacetylleucine (Aqneursa^TM^), an orally-administered amino acid derivative, is the only US Food and Drug Administration (FDA) approved monotherapy for the rare genetic lysosomal storage disease (LSD), Niemann-Pick disease type C (NPC) [1–3], conferring rapid symptomatic relief [4] as well as long-term disease-modifying effects [5]. The parent molecule, L-leucine, is a zwitterion at physiological pH, and is transported into cells by the easily saturable transporter L-type amino-acid transporter (LAT1). Addition of the acetyl moiety to L-leucine confers a net negative charge to the molecule at physiological pH, allowing levacetylleucine to be taken up into cells by high-capacity monocarboxylate transporters (MCTs), which are ubiquitously expressed, thereby delivering the drug to all tissues, including the central nervous system, by readily crossing the blood-brain barrier [6]. Inside cells, levacetylleucine enters enzyme-controlled pathways that correct metabolic dysfunction and enhance energy (ATP) production, which also leads to an improvement in lysosomal function [7,8]. This has multiple consequential effects: mitochondrial and lysosomal function are intrinsically linked, and interact at membrane contact sites [9] and the normalization of energy metabolism improves lysosomal function, leading to a reduction in the storage of unesterified cholesterol and sphingolipids [8]. In addition, levacetylleucine corrects aberrant membrane contact sites in NPC patient cells where there is a deficit of endoplasmic reticulum (ER)-lysosomal contacts for efficient lipid transfer [10] and a concomitant inappropriate gain of mitochondrial-ER contacts with resultant mitochondrial lipid accumulation [11].

In various animal models, levacetylleucine treatment shows a slowing of neurodegeneration and also leads to a dampening of neuroinflammation, consistent with the drug’s neuroprotective effects [8,12]: the latter was also shown in patients with NPC [13]. Due to its multi-modal mechanism of action, levacetylleucine has potential and is being developed for a range of rare and common neurodegenerative and neurodevelopmental disorders.

Niemann-Pick disease type C is a rare, debilitating, pre-maturely fatal disorder affecting 1/100,000 live births [14]. NPC is caused by mutations in either the *NPC1* or *NPC2* genes. The proteins they encode, NPC1 or NPC2, respectively, function cooperatively to facilitate lipid transport from the lysosome to the ER [10]. The NPC1 protein is a transmembrane protein involved in lipid transport, whilst NPC2 is a soluble luminal lysosomal protein thought to be involved in transferring lipids to NPC1 [15]. NPC gene dysfunction leads to the accumulation of multiple classes of lipids in lysosomes, causing lysosomal enlargement, disruption of intracellular lipid trafficking and autophagy, dysregulation of mTOR signalling, reduction in lysosomal Ca^2+^ storage and release, perturbation of energy metabolism and mitochondria function, and ultimately cell death [16]. Although all organs are affected, including fibroblasts, neurons are particularly susceptible with symptoms manifesting as impaired motor function and cognition.

Recently, it has been demonstrated that small molecule activation of transcription factor EB (TFEB) in NPC1^-/-^ cells, enhances lysosomal clearance [17]. TFEB, a key transcription factor affecting lysosomes, orchestrates the expression of the coordinated lysosomal expression and regulation or CLEAR network of genes promoting the expression of genes required for autophagosome formation, lysosome biogenesis, and lysosomal function and exocytosis [18]. TFEB contains basic helix-loop-helix-leucine zipper domains (bHLH-Zip) and belongs to the microphthalmia MiT-TFE family of transcription factors [19], and is highly expressed in the CNS [20]. In its inactive phosphorylated state, TFEB resides in the cytoplasm; upon dephosphorylation it dissociates from 14-3-3 proteins and translocates to the nucleus to regulate gene expression [15]. Dysregulation of TFEB activity is implicated in various neurodegenerative and neurodevelopmental diseases [21]. For instance, previous studies have demonstrated that targeting the TFEB pathway has neuroprotective effects in various *in vivo* or *in vitro* models of Alzheimer’s disease. Hence, small-molecule TFEB activators or heterologously overexpressed TFEB may promote lysosomal function and autophagic flux and may prevent, obstruct, or reverse the pathogenesis of neurodegenerative diseases [22].

In view of its clinical effects on a broad range of NPC disease manifestations downstream of a compromised lysosomal-mitochondrial axis, we investigated the role of levacetylleucine in modulating TFEB activity, the key regulator of lysosome biogenesis. Surprisingly, we find that a major effect of levacetylleucine is to normalize TFEB activity, including to reduce TFEB activation in *NPC1*^*-/-*^ cells where TFEB activity is already elevated.

## Materials and methods

### Cell culture and transfection

HeLa cells were cultured in DMEM supplemented with 10% v/v FCS, 2 mM glutamine, 100 U/ml penicillin and 100 µg/ml streptomycin, at 37^o^C under 5% CO_2_. Cells were trypsinized and seeded onto CellView Slides (Greiner Bio-One).1-2 days after sub-culturing, cells were transiently transfected with JetPEI reagent in a 5:2 ratio with DNA. Per well, cells were transfected with 100 ng TFEB tagged on its C-terminus with either EGFP (Addgene plasmid # 38119) or mScarlet3 (produced in-house) for 4–6 hrs. Transfection medium was removed and replaced with fresh DMEM with or without agents and incubated overnight at 37^o^C. The next day, TFEB-EGFP-expressing cells were loaded for 1h at 37^o^C with NucSpot® Live 650 Nuclear Stain (Biotium) in the continued presence of drugs as required. Cells were then transferred into extracellular medium (ECM, mM: 121 NaCl, 5.4 KCl, 0.8 MgCl_2_, 1.8 CaCl_2_, 6 NaHCO_3_, 25 HEPES, 10 Glucose) that maintained the overnight treatment reagents and these live cells were imaged immediately.

The NPC1 KO HeLa cell line (ex2 NPC1-KO) was generated by the CRISPR-Cas9 technique and verified, as previously described. [23]

Cells expressing TFEB-mScarlet3 were treated with similar reagent protocols but could be fixed with 4% PFA (in PBS) after reagent treatments. After fixation and permeabilization (0.1% Triton X-100 in PBS for 15 mins), nuclei were labelled with NucSpot® Live 488 Nuclear Stain (Biotium) for 10 mins in PBS and imaged within 2 days.

### Western blotting

WT and NPC1^-/-^ HeLa cells were lysed in RIPA Buffer (Cell Signalling Technology) supplemented with protease inhibitor cocktail (Roche). Protein concentrations of the spun lysates were measured using the bicinchoninic acid method (BCA Protein Assay Kit, Pierce). 20 µg of cell lysate were separated on 4–12% SDS–PAGE gel (Thermo Fisher Scientific), transferred to PVDF membranes followed by blocking with 5% dried milk in 0.1% Tween 20/PBS solution. Membranes were then probed with primary polyclonal anti-NPC1 (NB400−148, Novus) antibody, secondary antibodies conjugated to HRP and developed with ELC substrate (Thermo Fisher Scientific). Chemiluminescent signal was detected using an Universal II hood (BioRad).

### Microscopy

Cells were imaged at room temperature using a Nikon A1R laser-scanning confocal equipped with a Plan ApoVC 20x DIC N2 (NA: 0.75) or Plan Fluor 40x oil DIC H N2 (NA: 1.3) objective. In Channel-Series mode, green, red or far-red fluorophores were alternately excited (ex/em): 488/525 nm, 561/595 nm, and 640/700 nm respectively.

### Nuclear translocation analysis

To quantify the translocation of fluorescent TFEB from the cytoplasm to the nucleus, we analysed the Pearson’s Correlation Coefficient between TFEB and the orthogonal nuclear stain in single cells. Cells with a cytoplasmic location have negative coefficients, whereas nuclear translocation is reflected by positive coefficients. Cells with nuclear TFEB (partial or completely translocated) were defined as having a Pearson’s coefficient >0, and the number of cells satisfying this criterion expressed as a percentage of the total number.

### Reverse transcription quantitative PCR (RT-qPCR)

Total RNA was extracted from wild-type HeLa cells using a RNeasy Plus Mini kit (Qiagen); quality and quantity were assessed with a NanoDrop spectrophotometer (Thermo Fisher Scientific). cDNA was prepared using an iScript cDNA synthesis kit in a MyCycler Thermal Cycler (both Bio-Rad). qPCR was then performed with a CFX96 Real-Time PCR instrument (Bio-Rad) using PowerUp SYBR Green (Applied Biosystems) and the primer pairs specified in Table 1. Primers were obtained from OriGene except for the β-actin primer pair, which was sourced via PrimerBank (ID 4501885a). Threshold cycle (C_t_) values were normalised to the reference gene β-actin using the comparative C_t_ method, on a scale whereβ-actin expression equals 10,000 units.

**Table 1:** Primer sequences for RT-qPCR.

Gene	Forward Sequence	Reverse Sequence
*ACY1* (aminoacylase 1)	CCTACACTCTCCTCCATCTTGC	CCTGGCATAGATGTAGCCCTCA
*ACTB* (β-actin)	CATGTACGTTGCTATCCAGGC	CTCCTTAATGTCACGCACGAT
*SLC16A1* (MCT1)	TTGTTGGTGGCTGCTTGTCAGG	TCATGGTCAGAGCTGGATTCAAG
*SLC16A7* (MCT2)	TGCTGGCTGTTATGTACGCAGG	GCCAACACCATTCCAAGACAGC
*SLC16A8* (MCT3)	TGCAGTTCGAGGTGCTCATGGC	GTTCTTCAACACATCCACCAGGC
*SLC16A3* (MCT4)	CCACAAGTTCTCCAGTGCCATTG	CGCCAGGATGAACACGTACATG

### Immunofluorescent labelling of endogenous TFEB

Cells were fixed with 4% PFA (in PBS), permeabilised (0.1% Triton X-100 in PBS for 15 min) and blocked (5% goat serum in PBS for 30 min). Antibody incubations were performed in PBS/0.01% Triton X-100/5% goat serum. The primary antibody anti-TFEB (Cell Signaling Technology, #4240) was used at 1:500 and a goat anti-rabbit IgG conjugated to Alexa 546 was used as the secondary antibody. Nuclei were counter-labelled with NucSpot® Live 488 Nuclear Stain (Biotium) for 10 mins in PBS.

### In-cell western for detection of LAMP1 and TFEB

Cells were seeded into a 96-well flat-bottomed µ-clear black plate (Greiner Bio-One). Following treatment with 2 mM NALL for 22 hours at 37^o^C, 5% CO_2_, cells were fixed with 4% PFA in PBS and permeabilized with 0.1% Triton X-100. Following block in Odyssey® blocking buffer (LICORbio), cells were incubated with primary antibodies. For labelling of LAMP-1, cells were incubated with LAMP1 (H4A3) mouse monoclonal (DSHB at The University of Iowa) and detected using IRDye 800CW goat anti-mouse IgG (LICORbio); the cells were co-stained with CellTag 520 Stain. For TFEB detection, cells were incubated with TFEB (E4I8R) mouse monoclonal (Cell Signaling Technology, #91767), for total TFEB levels and Phospho-TFEB (Ser122) (E9M5M) rabbit monoclonal (Cell Signaling Technology, #87932) and were detected using IRDye® 680RD donkey anti-mouse IgG and IRDye® 800CW donkey anti-rabbit IgG polyclonals (LICORbio) respectively. Cells were scanned using an Odyssey M Imaging System in the 700 and 800 nm channels and analysed using Empiria Studio (LICORbio).

## Results

### HeLa cells express the monocarboxylate transporters and aminocyclase 1 enzyme required for levacetylleucine action

We have previously proposed that levacetylleucine acts as a pro-drug to generate high concentrations of L-leucine in cells since it is a substrate for MCT transporters [6]. Therefore, in the first series of experiments we investigated whether HeLa cells express the key components of the levacetylleucine transport mechanisms previously proposed. We found that HeLa cells express the transporter system and acylase that are thought necessary for a response to levacetylleucine. Expression patterns of monocarboxylate transporters (MCT1, MCT2, MCT3, MCT4) and the aminoacylase 1 enzyme (ACY1) were determined by RT-qPCR (primer sequences in Table 1). Expression was normalized to β-actin, on a scale where β-actin expression equals 10,000 arbitrary units. We show that HeLa cells contain transcribed mRNA for three monocarboxylate transporter types, including MCT1 1, (S1 Fig) as well as an acylase shown to hydrolyze N-acetyl groups from amino acids [24,25]. The resulting L-leucine enters metabolic pathways to increase ATP synthesis, but also acts to clear stored lipids from lysosomes [7].

### Levacetylleucine promotes rapid activation of TFEB in WT HeLa cells

**Fig 1:**
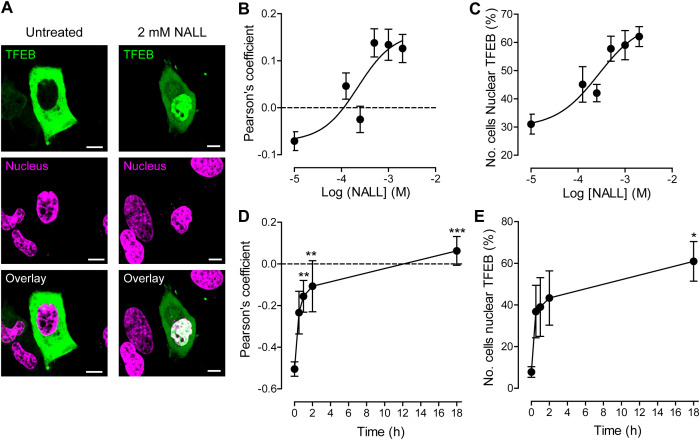
NALL induces nuclear translocation of TFEB. HeLa cells were transfected with TFEB-EGFP (A-C) or TFEB-mScarlet3 (D,E) and then incubated with NALL at different concentrations or times. Cells were then counter-stained with the NucSpot 488 (D,E) or NucSpot 650 (A-C) to label the nucleus and TFEB translocation quantified by colocalization with NucSpot. (A) Single-cell images of TFEB-EGFP (green) and NucSpot 650 (magenta). (B,C) Concentration-response of HeLa treated for 18 hrs with NALL. Nuclear localization is plotted as either the Pearson’s coefficient (B) or derived as the percentage of cells with nuclear TFEB (C). (D,E) Time course of the effect of 2 mM NALL upon TFEB, either expressed as the Pearson’s coefficient (D) or the percentage of cells with nuclear TFEB (E). The dotted lines (B,D) highlight a correlation coefficient of zero used for thresholding the percentage of nuclear cells. Data are expressed as the mean ± SEM of 179-215 cells, and significance determined by ANOVA test, with significance depicted as * *p* < 0.05, ** *p* < 0.01, or *** *p* < 0.001 compared to respective control, 0 mM (B,C) or t = 0 (D,E).

To test for a direct effect of the drug on lysosomal function, we examined the effect of treating HeLa with levacetylleucine on the translocation of TFEB-GFP from cytoplasm to nucleus, a hallmark for the activation of this transcription factor [26]. We found that extracellular incubation of cells with levacetylleucine (NALL, 2 mM) caused the appearance of TFEB-GFP in the nucleus during an 18-hour incubation (Fig 1A). The effect was concentration-dependent with effects seen at the sub-millimolar range (Fig 1B, C), which coincides with plasma concentrations observed in mice dosed with therapeutic concentrations of levacetylleucine [27].

**Fig 2:**
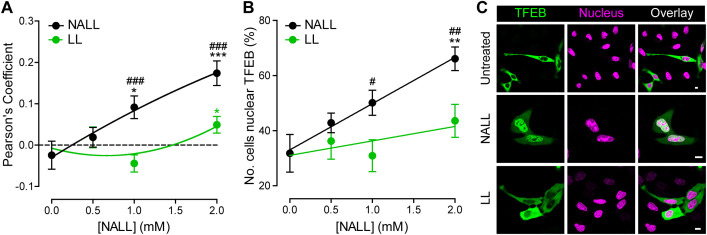
NALL induces TFEB translocation more efficaciously than does L-leucine. TFEB-EGFP translocation in HeLa cells incubated for 18 hours with different concentrations of either NALL (black) or L-Leucine (LL, green). Translocation is expressed either as the Pearson’s correlation coefficient with NucSpot 650 (A) or as the percentage of cells with nuclear TFEB (B). Data are expressed as the mean ± SEM of 104-161 cells, and significance determined by nonparametric ANOVA test (A) or unpaired t-test (B), with significance depicted as * *p* < 0.05, ** *p* < 0.01, or *** *p* < 0.001 compared to the vehicle control, i.e., 0 mM. Comparing NALL and LL: #, *p* < 0.05; ###, *p* < 0.001. (C) Single-cell images of TFEB-EGFP (green) and NucSpot 650 (magenta) in untreated, 2 mM NALL- or 2 mM LL-treated HeLa, where the two co-localize is shown as white, scale bar = 10 µm.

The onset of action of levacetylleucine was rapid, with half-maximal effect seen within 60 min (Fig 1 D, E) during continuous application of the drug. Importantly, this effect was not shared by L-leucine over a similar concentration range (Fig 2), which is the major metabolite of levacetylleucine through the action of amino acid acylases [28], demonstrating that this effect required the acetyl form of leucine (as in levacetylleucine). This effect is likely only be transient since levacetylleucine is converted to L-leucine by cellular acylases [25,27].

### Levacetylleucine (N-acetyl-L-leucine) is the superior enantiomer for inducing TFEB activation

**Fig 3:**
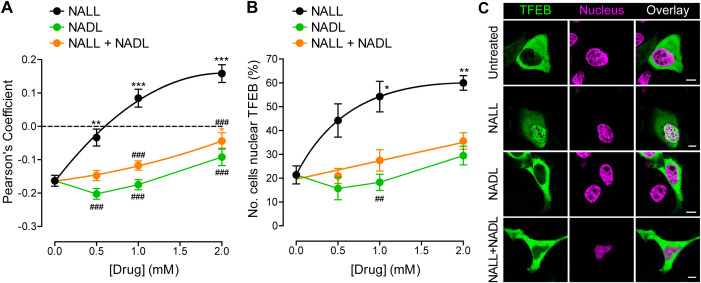
NALL is the most efficacious enantiomer to evoke TFEB translocation. TFEB-EGFP translocation in HeLa cells incubated for 18 hours with different concentrations of each enantiomer: the L-form (NALL, black), D-form (NADL, green) or the racemic mixture of both (orange). Translocation is expressed either as the Pearson’s correlation coefficient with NucSpot 650 (A) or as the percentage of cells with nuclear TFEB (B). Data are expressed as the mean ± SEM of 153-265 cells, and significance determined by nonparametric ANOVA test, with significance denoted by * *p* < 0.05, ** *p* < 0.01, or *** *p* < 0.001 compared to vehicle control, i.e., 0 mM. Comparing NALL and NADL or racemic mixture, using a nonparametric ANOVA test: ##, *p* < 0.01; ###, *p* < 0.001. (C) Single-cell images of TFEB-EGFP (green) and NucSpot 650 (magenta) in untreated, 2 mM NALL-, 2 mM NADL- and 1 mM NALL + 1 mM NADL-treated HeLa; co-localization (white), scale bar = 10 µm.

Since the racemic mixture, N-acetyl-D-L-leucine (Tanganil) has also been suggested to have beneficial effects in lysosomal storage diseases [7], we examined the effects of the separate enantiomers levacetylleucine and N-acetyl-D-leucine as well as the racemate N-acetyl-D-L-leucine on TFEB translocation to the nucleus (Fig 3). While levacetylleucine caused a robust translocation (activation) of TFEB into the nucleus, the D-enantiomer, N-acetyl-D-leucine, had no effect. Remarkably, the racemate N-acetyl-D-L-leucine also failed to activate TFEB translocation, indicating that the presence of the D-enantiomer in the racemic mixture antagonises the effect of the pharmacologically active L-enantiomer, levacetylleucine.

### Levacetylleucine normalises TFEB distribution in NPC1 disease models

**Fig 4:**
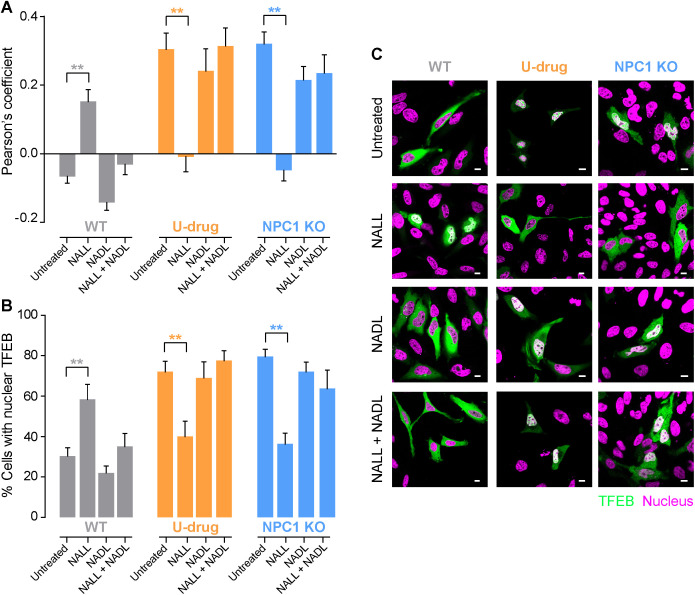
TFEB is constitutively active in NPC and reduced by NALL, but not by NADL or the racemic NADLL. WT HeLa, WT HeLa treated with 2 µg/ml U18666A (U-drug) for 22 hours and NPC1 KO HeLa were transfected with TFEB-mScarlet3 and then incubated with 2 mM NALL, 2 mM NADL, or the racemic mixture of both (1 mM NALL + 1 mM NADL) for 22 hours. Cells were counter-stained with NucSpot 488 to label the nucleus and TFEB translocation quantified by colocalization with NucSpot. Nuclear localization is plotted as either the Pearson’s coefficient (A) or derived as the percentage of cells with nuclear TFEB (B). Data are expressed as the mean ± SEM of 101-321 cells, and significance determined by ANOVA test: ** *p* < 0.01. (C) Images of TFEB-mScarlet3 (green), NucSpot 488 (magenta) in single-cells treated as in (A, B); co-localization (white), scale bar = 10 µm.

We next assessed the effect of Levacetylleucine on TFEB distribution in NPC1 disease models: in CRISPR-Cas9-edited NPC1 knockout (NPC1^-/-^) HeLa cells (S2 Fig) and in pharmacological U18666A (U-drug) treated HeLa cells. In contrast to the predominantly cytoplasmic distribution in untreated, wild-type HeLa cells, we found that in NPC1^-/-^ cells there was significantly higher nuclear TFEB localisation (Fig 4), e.g., the overactivation of TFEB activity. We also found that incubation of wild-type cells with the NPC1 small molecule inhibitor, U18666A [29] increased the nuclear translocation by a similar degree to 2 mM levacetylleucine (Fig 4).

In *NPC1*^*-/-*^ cells and U18666A-administered wild-type cells, treatment with levacetylleucine reduced nuclear localization to a level approximating that seen with treatment of wild-type cells with levacetylleucine (2 mM). This down-regulation of TFEB in cells where it was already upregulated reflected a normalization of TFEB localisation, and was also stereo-specific as for TFEB activation in wildtype cells (Fig 3), in that the D-enantiomer or racemate had no effect (Fig 4).

### Levacetylleucine normalises endogenous TFEB distribution in NPC1 disease models by promoting Serine 122 phosphorylation

While in the above experiments, we employed an heterologously expressed fluorescent-TFEB probe to assess its modulation by levacetylleucine, in subsequent experiments, we investigated the effects of levacetylleucine on endogenous TFEB activation state in two ways. The first approach was to label TFEB in cells with an anti-TFEB monoclonal antibody to assess its distribution between the cytoplasm and nucleus. The second approach was to probe the phosphorylation state of TFEB which also reflects its activation state with a specific phospho-specific antibody for TFEB. Phosphorylation of TFEB at Serine 122 (Ser122) acts as a molecular switch for controlling cellular stress responses in the regulation of lysosomal biogenesis and autophagy. Under nutrient-replete conditions, mTORC1 (mechanistic target of rapamycin complex) directly phosphorylates TFEB at Ser122, contributing to its sequestration in the cytoplasm and inhibition of its activity [30].

**Fig 5:**
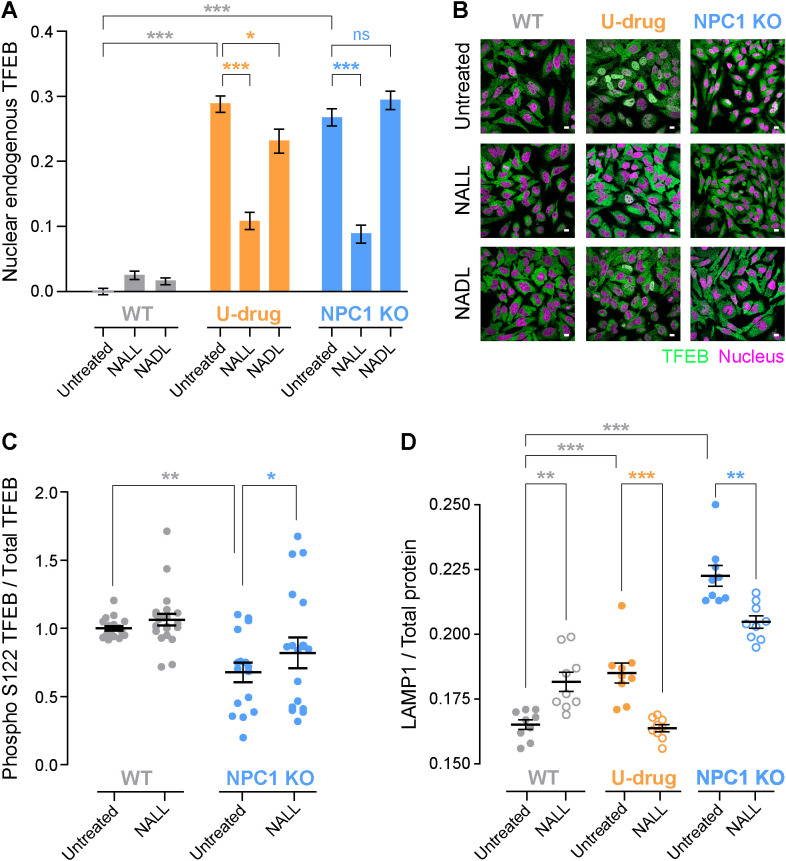
NALL enhances translocation of endogenous TFEB from the nucleus in NPC1 disease model cells. (A) WT, WT treated with 2 µg/ml U18666A (U-drug) for 22 hours and NPC1 KO HeLa were incubated with 2 mM NALL or 2 mM NADL for 22 hours. Endogenous TFEB was labelled with an anti-TFEB antibody and detected using a fluorescently coupled secondary antibody. Cells were counter-stained with the NucSpot 488 to label the nucleus and TFEB translocation quantified by colocalization with NucSpot 488. Data are expressed as the mean ± SEM of 133-500 cells normalized to the WT untreated control, and significance determined by two-way ANOVA, ***p < 0.001, *p < 0.05, ns (non-significant). (B) Single-cell images of endogenous TFEB (green) and NucSpot 488 (magenta) in untreated, 2 mM NALL-and 2 mM NADL-treated cells; co-localization (white), scale bar = 10 µm. (C) Treatment of WT and NPC1 KO HeLa with 2 mM NALL for 22 hours increases phosphorylation of endogenous TFEB at Serine 122 (S122). Data are expressed as the mean ± SEM of S122 phosphorylation normalised to the total TFEB, as determined by In-Cell Western. Each circle represents an individual sample (N = 4, n = 16-23). Significance determined by one-way ANOVA: ** P < 0.01, *** *p* < 0.001. (D) WT, WT treated with 2 µg/ml U18666A (U-drug) and NPC1 KO HeLa were incubated with 2 mM NALL for 44 hours, then processed for In-Cell Western analysis: endogenous LAMP1 was labelled with an anti-LAMP1 antibody and stained with CellTag520 (for normalization to total protein). Data are expressed as the mean ± SEM of 9 replicates where each circle represents an individual sample. Significance determined by two-way ANOVA, *** *p* < 0.001, ** *p* < 0.01.

First in terms of nuclear versus cytoplasmic localization of TFEB, in wildtype cells treated with the NPC1 inhibitor U18666A or in NPC1^-/-^ cells, there was a greater nuclear localization when compared to untreated wildtype cells (Fig 5A, B). When wildtype cells were treated with levacetylleucine, there was no significant change in endogenous TFEB localization. Whilst levacetylleucine induces a clear and pronounced translocation of overexpressed TFEB to the nucleus (Figs 1-4), it does not significantly induce translocation of endogenous TFEB. This discrepancy may be due to the difference in quantification methods comparing endogenous TFEB by antibody labelling with TFEB-EGFP (or mScarlet3) overexpression which provided a wider dynamic range which makes even subtle translocation of TFEB appear stronger. The higher TFEB-EGFP abundance amplifies the visible response and this can make levaceylleucine appear more potent in the overexpression system. Furthermore, endogenous TFEB is often expressed at relatively low levels it may be difficult to distinguish small localization changes. With this in mind, it is even more remarkable that treatment of either U18666A-administered cells or NPC1^-/-^ cells with levacetylleucine caused a dramatic, significant decrease in nuclear endogenous TFEB (Fig 5A, B). Whilst treatment with the D-form, NADL, was predominantly without effect on endogenous TFEB nuclear localization (Fig 5A, B).

Similarly, probing the status of the Ser122 phosphorylation site on endogenous TFEB in NPC1^-/-^ cells, showed a significant reduction in Ser122 phosphorylation, indicating activation of TFEB (i.e., a more nuclear localisation) in the NPC1 disease cells compared to wildtype cells (Fig 5C). Upon treatment with levacetylleucine (2 mM) there was an increase in Ser122 phosphorylation in NPC1^-/-^ cells (Fig 5C) which correlates with TFEB translocation to the cytosol upon levacetylleucine-treatment (Fig 5A, B). This would be consistent with levacetylleucine acting as a prodrug to yield a rise in intracellular L-leucine concentrations [6] to activate the mTOR complex resulting in the phosphorylation and cytoplasmic localization of TFEB resulting in its inactive state [30].

To assess if the activation of TFEB and its nuclear translocation has functional consequences, we showed that the expression of the major lysosomal transmembrane protein, LAMP1, whose gene has a CLEAR sequence in its promoter region which affords regulation of its expression by TFEB [31], is significantly increased in U18666A-administered HeLa or in NPC1^-/-^ cells (Fig 5D). This is indicative of an increased nuclear TFEB localization (Fig 5A, B), which in turns promotes translation of the LAMP1 gene. Upon incubation of wildtype HeLa with levacetylleucine, the expression of LAMP1 is also increased, again indicative of an increased nuclear localization of TFEB (Figs 1-5). Furthermore, treatment of U18666A-administered HeLa or in NPC1^-/-^ cells with levacetylleucine (2 mM) significantly reduced the expression of LAMP1 (Fig 5D), in line with levacetylleucine normalizing the TFEB balance between nuclear and cytosol localization and thence expression of lysosomal proteins.

## Discussion

The study establishes that Levacetylleucine (Aqneursa™) acts as a direct modulator of lysosomal regulation and function by influencing the translocation of TFEB, the master regulator of the CLEAR network, in a cellular model of NPC. A critical finding is the stereospecific nature of this response; only the L-enantiomer (levacetylleucine) is pharmacologically active, whereas the D-enantiomer and the racemate fail to induce TFEB translocation. This suggests that the D-enantiomer may even act antagonistically, highlighting why the purified L-enantiomer is the preferred therapeutic form.

The most striking result is the drug’s bidirectional effect on TFEB activity, which appears to restore cellular homeostasis regardless of the starting state. In wild-type cells, levacetylleucine promotes TFEB activation and nuclear localisation, leading to increased expression of lysosomal proteins like LAMP1. In contrast in NPC1^-/-^ cells, we show that TFEB is already over-activated (likely due to chronic lysosomal stress induced by lipid storage) levacetylleucine effectively reduces nuclear TFEB, bringing activity levels back toward a normal physiological balance. This is most strongly seen using the fluorescently tagged TFEB protein, heterologously expressed. For endogenous TFEB, there is little effect on normal TFEB activity/localization in wildtype cells, but a strong inhibitory effect in disease cells, again highlighting the normalization effect of levacetylleucine treatment.

The efficacy of levacetylleucine is tied to its superior cellular uptake via monocarboxylate transporters (MCTs). Unlike its parent molecule, L-leucine, which relies on the easily saturable LAT1 transporter, levacetylleucine leverages high-capacity MCTs to reach therapeutic concentrations within the cell. Once inside, levacetylleucine is metabolised by acylases into L-leucine, which then fuels mitochondrial ATP production and corrects metabolic dysfunction [6]. The study proposes a multi-step model where levacetylleucine initially inhibits mTORC1, triggering lysosomal release and TFEB dephosphorylation (activation), followed by a metabolic phase where the generated L-leucine reactivates mTOR and enhances bioenergetics. Although TFEB is best known as a target of mTORC1, there are other mTORC1-independent mechanisms that regulate its localization and activity, of which levacetylleucine may affect. These include dephosphorylation of TFEB by calcineurin, or the phosphorylation of TFEB directly without involving mTORC1 by ERK2 or GSK3β or AKT. Whilst AMPK signalling can activate TFEB both dependently and independently of mTORC1.

The ability of levacetylleucine to correct aberrant TFEB activity at clinically relevant concentrations provides a further potential mechanistic explanation for its observed neuroprotective and disease-modifying effects in NPC, and other neurological disease patients. By reducing the storage of unesterified cholesterol and sphingolipids and dampening neuroinflammation, levacetylleucine addresses the core pathologies of NPC. Furthermore, because TFEB dysregulation is a common feature across many neurodegenerative and neurodevelopmental disorders—including Alzheimer’s—the findings suggest that levacetylleucine’s therapeutic potential may extend far beyond rare lysosomal storage diseases.

While Transcription Factor EB (TFEB) is a crucial regulator of lysosomal biogenesis and autophagy, promoting cellular clearance in neurodegenerative diseases, excessive or constitutive activation of TFEB can be detrimental to cells and neurons. TFEB overactivation often leads to “lysosomal saturation,” where the generation of new lysosomes fails to keep pace with autophagosome formation, resulting in the accumulation of autophagic vacuoles rather than their degradation [32]. Sustained TFEB induction can cause an uncontrolled increase in lysosomal numbers and enzymatic activity, which may result in lysosomal membrane permeabilization (LMP) [33]. This leakage of cathepsins into the cytosol initiates apoptosis [34]. Furthermore, chronic TFEB activity can exhaust the cell’s metabolic resources. By over-promoting catabolism, cells may experience “autophagic burnout,” where the excessive degradation of healthy organelles, including mitochondria, leads to energy failure (ATP depletion) [35].

This impairment of autophagic flux can lead to cellular, and specifically neuronal, toxicity rather than protection. Studies indicate that excessive ROS levels resulting from metabolic stress can combine with high TFEB activity to cause lysosomal dysfunction, autophagic failure, and subsequent cell death [36]. Furthermore, while moderate activation helps, TFEB overexpression has been shown to be toxic under high levels of heavy metal exposure (such as copper), increasing oxidative stress and mitochondrial damage [37].

In the brain, excessive TFEB activity may disrupt cellular homeostasis, as its high expression can be linked to cancer proliferation (e.g., renal cell carcinoma). In the context of neurons, while balancing TFEB is therapeutic, uncontrolled overactivation can lead to detrimental metabolic shifts and, in certain cases, enhanced accumulation of toxic Serine129-phosphorylated α-synuclein, a key marker of neurotoxicity in Parkinson’s disease models [38]. Consistent with this, a recent study has shown that levacetylleucine lowers a-synuclein levels in human iPSC derived neurons [39].

Of note, TFEB activation has recently been proposed as a mechanism of action for another newly approved NPC drug, arimoclomol [40]. However, despite the findings that TFEB is constitutively active in NPC, arimoclomol had the limited ability to only activate TFEB as opposed to normalise or down-regulate TFEB, which may be potentially problematic. The same caution could be applied to a direct TFEB activator recently proposed as a potential new therapy for NPC [17].

We have previously also shown that levacetylleucine has a biphasic effect normalizing mitochondrial function; levacetylleucine rebalances energy metabolism, irrespective of whether the pathophysiology of the disease is characterized by insufficient or excessive Krebs cycle flux, changing the expression levels of key enzymes that regulate these key pathways [7,8].

**Fig 6:**
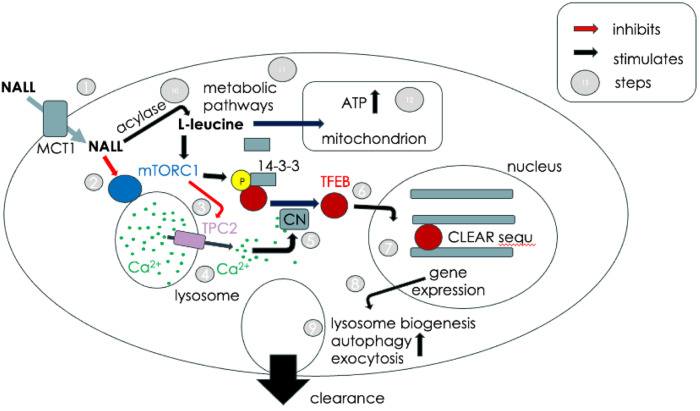
Scheme for the mechanisms of action of levacetylleucine (NALL) to enhance lysosomal and mitochondrial function in WT cells. **Step 1** Levacetylleucine (NALL) is transported into cells via the MCT transporter family. **Step 2** Levacetylleucine inhibits the activity of the mTORC complex and reduces TFEB phosphorylation. **Step 3** Inhibition of mTOR relives its inhibition on Ca^2+^ release channels expressed in lysosomes leading to Ca^2+^ release by TPC2 and/or TRPML1 (**Step 4**). **Step 5** Lysosomal Ca^2+^ release activates calcineurin (CN) to dephosphorylate TFEB and remove its ability to bind 14-3-3 proteins allowing it to translocate to the nucleus (**Step 6**). **Step 7** TFEB bind to CLEAR gene promoters and activates lysosomal and autophagic gene expression. **Step 8** This increases lysosomal biogenesis, autophagy and lysosomal exocytosis leading to cellular clearance of stored lysosomal material (**Step 9**). **Step 10** Levacetylleucine is metabolised to L-leucine that reactivates mTOR resulting in TFEB inactivation through phosphorylation. **Step 11** L-leucine enters metabolic pathways, where it increases mitochondrial ATP production (**Step 12**). In NPC1^-/-^ cells, levacetylleucine has the opposite effect in that it now reduces the activity of TFEB that is constitutively enhanced in this model of NPC possibly due to the reactivation of mTORC1 by L-leucine.

Our findings here that the “normalisation” of TFEB activity by levacetylleucine suggests that the drug does not simply “turn on” a pathway but rather recalibrates the lysosomal-mitochondrial axis, as depicted in Fig 6. This has widespread implications for this drug in restoring normal cellular homeostasis, [8] reinforcing its promise as a new therapy for broad neurodegenerative and neurodevelopmental disease management.

## Supporting information

S1 Fig Expression patterns of monocarboxylate transporters (MCT1, MCT2, MCT3, MCT4) and the aminoacylase 1 enzyme (ACY1). These were determined by RT-qPCR. Expression was normalised to β-actin, on a scale whereβ-actin expression equals 10,000 arbitrary units. Data are shown as the mean (bars) of two biological replicates (symbols) on a logarithmic scale. MCT3 expression was not detected.
(DOCX)

S2 Fig Western Blot of NPC1 protein in wild-type and NPC1^-/-^ cells. An NPC1 KO HeLa cell line (exon 2 NPC1-KO) was generated by the CRISPR-Cas9 technique and verified, as previously described [23]. Cell lysates were probed with primary polyclonal anti-NPC1 antibody, secondary antibodies conjugated to HRP and developed with ELC substrate.
(DOCX)

## Abbreviations

ATP: adenosine triphosphate
CLEAR network: coordinated lysosomal expression and regulation network
ER: endoplasmic reticulum
FDA: Food and Drug Administration (USA)
INN: International Nonproprietary Names
LAMP-1: Lysosome-associated membrane protein 1
LAT: L-type amino-acid transporter
LSD: lysosomal storage disease
MCT: monocarboxylate transporter
mTORC1: mammalian target of rapamycin complex 1
NPC: Niemann-Pick disease type C
NALL: N-acetyl-L-leucine
MCS: membrane contact site(s)
RBD: REM sleep behaviour disorder
TFEB: Transcription Factor EB
USAN: United States Adopted Name

## References

[1] Beninger P. Aqneursa (levacetylleucine). Clin Ther. 2024;46(12):1091–2. doi: 10.1016/j.clinthera.2024.10.011 39567327

[2] Mullard A. FDA approves first two drugs for rare Niemann-Pick disease. Nat Rev Drug Discov. 2024;23(11):804. doi: 10.1038/d41573-024-00162-9 39333713

[3] van Gool R, Al-Hertani W, Bodamer O, Upadhyay J. Levacetylleucine (N-acetyl-l-leucine) for Niemann-Pick disease type C. Trends Pharmacol Sci. 2025;:386–7. doi: 10.1016/j.tips.2025.02.003 40055076

[4] Bremova-Ertl T, Ramaswami U, Brands M, Foltan T, Gautschi M, Gissen P. Trial of N-Acetyl-l-Leucine in niemann-pick disease type C. N Engl J Med. 2024;390(5):421–31. doi: 10.1056/NEJMoa2310151 38294974

[5] Patterson MC, Ramaswami U, Donald A, Foltan T, Gautschi M, Gissen P, et al. Disease-modifying, neuroprotective effect of N-Acetyl-l-leucine in adult and pediatric patients with niemann-pick disease type C. Neurology. 2025;105(1):e213589. doi: 10.1212/WNL.0000000000213589 40513057 PMC12296777

[6] Churchill GC, Strupp M, Factor C, Bremova-Ertl T, Factor M, Patterson MC, et al. Acetylation turns leucine into a drug by membrane transporter switching. Sci Rep. 2021;11(1):15812. doi: 10.1038/s41598-021-95255-5 34349180 PMC8338929

[7] Kaya E, Smith DA, Smith C, Boland B, Strupp M, Platt FM. Beneficial effects of acetyl-DL-leucine (ADLL) in a mouse model of sandhoff disease. J Clin Med. 2020;9(4):1050. doi: 10.3390/jcm9041050 32276303 PMC7230825

[8] Kaya E, Smith DA, Smith C, Morris L, Bremova-Ertl T, Cortina-Borja M, et al. Acetyl-leucine slows disease progression in lysosomal storage disorders. Brain Commun. 2020;3(1):fcaa148. doi: 10.1093/braincomms/fcaa148 33738443 PMC7954382

[9] Martello A, Platt FM, Eden ER. Staying in touch with the endocytic network: The importance of contacts for cholesterol transport. Traffic. 2020;21(5):354–63. doi: 10.1111/tra.12726 32129938 PMC8650999

[10] Höglinger D, Burgoyne T, Sanchez-Heras E, Hartwig P, Colaco A, Newton J, et al. NPC1 regulates ER contacts with endocytic organelles to mediate cholesterol egress. Nat Commun. 2019;10(1):4276. doi: 10.1038/s41467-019-12152-2 31537798 PMC6753064

[11] Kiraly S, Martello A, Platt FM, Eden ER. N-Acetyl-l-Leucine (NALL) rescues inter-organelle communication in Niemann-Pick disease type-C patient cells. BioRxiv. 2025. doi: 10.1101/2025.04.27.650877

[12] Hegdekar N, Lipinski MM, Sarkar C. N-Acetyl-L-leucine improves functional recovery and attenuates cortical cell death and neuroinflammation after traumatic brain injury in mice. Sci Rep. 2021;11(1):9249. doi: 10.1038/s41598-021-88693-8 33927281 PMC8084982

[13] Dinkel L, Hummel S, Zenatti V, Malara M, Tillmann Y, Colombo A, et al. Myeloid cell-specific loss of NPC1 in mice recapitulates microgliosis and neurodegeneration in patients with Niemann-Pick type C disease. Sci Transl Med. 2024;16(776):eadl4616. doi: 10.1126/scitranslmed.adl4616 39630885

[14] Tifft CJ. N-Acetyl-l-leucine and neurodegenerative disease. N Engl J Med. 2024;390(5):467–70. doi: 10.1056/NEJMe2313791 38294981

[15] Rosenbaum AI, Maxfield FR. Niemann-Pick type C disease: molecular mechanisms and potential therapeutic approaches. J Neurochem. 2011;116(5):789–95. doi: 10.1111/j.1471-4159.2010.06976.x 20807315 PMC3008286

[16] Platt FM, d’Azzo A, Davidson BL, Neufeld EF, Tifft CJ. Lysosomal storage diseases. Nat Rev Dis Primers. 2018;4(1):27. doi: 10.1038/s41572-018-0025-4 30275469

[17] Du K, Chen H, Pan Z, Zhao M, Cheng S, Luo Y, et al. Small-molecule activation of TFEB alleviates Niemann-Pick disease type C via promoting lysosomal exocytosis and biogenesis. Elife. 2025;13:RP103137. doi: 10.7554/eLife.103137 40184172 PMC11970905

[18] Napolitano G, Ballabio A. TFEB at a glance. J Cell Sci. 2016;129(13):2475–81. doi: 10.1242/jcs.146365 27252382 PMC4958300

[19] Puertollano R, Ferguson SM, Brugarolas J, Ballabio A. The complex relationship between TFEB transcription factor phosphorylation and subcellular localization. EMBO J. 2018;37(11):e98804. doi: 10.15252/embj.201798804 29764979 PMC5983138

[20] Cortes CJ, La Spada AR. TFEB dysregulation as a driver of autophagy dysfunction in neurodegenerative disease: Molecular mechanisms, cellular processes, and emerging therapeutic opportunities. Neurobiol Dis. 2019;122:83–93. doi: 10.1016/j.nbd.2018.05.012 29852219 PMC6291370

[21] Chen H, Gong S, Zhang H, Chen Y, Liu Y, Hao J, et al. From the regulatory mechanism of TFEB to its therapeutic implications. Cell Death Discov. 2024;10(1):84. doi: 10.1038/s41420-024-01850-6 38365838 PMC10873368

[22] Gu Z, Cao H, Zuo C, Huang Y, Miao J, Song Y, et al. TFEB in Alzheimer’s disease: From molecular mechanisms to therapeutic implications. Neurobiol Dis. 2022;173:105855. doi: 10.1016/j.nbd.2022.105855 36031168

[23] Tharkeshwar AK, Trekker J, Vermeire W, Pauwels J, Sannerud R, Priestman DA, et al. A novel approach to analyze lysosomal dysfunctions through subcellular proteomics and lipidomics: the case of NPC1 deficiency. Sci Rep. 2017;7:41408. doi: 10.1038/srep41408 28134274 PMC5278418

[24] Bloch K, Borek E. Biological acetylation of natural amino acids. J Biol Chem. 1946;164:483. 20989507

[25] BlocH K, RittenberG D. The metabolism of acetylamino acids. J Biol Chem. 1947;169(3):467–76. 20259079

[26] Settembre C, Zoncu R, Medina DL, Vetrini F, Erdin S, Erdin S, et al. A lysosome-to-nucleus signalling mechanism senses and regulates the lysosome via mTOR and TFEB. EMBO J. 2012;31(5):1095–108. doi: 10.1038/emboj.2012.32 22343943 PMC3298007

[27] Churchill GC, Strupp M, Galione A, Platt FM. Unexpected differences in the pharmacokinetics of N-acetyl-DL-leucine enantiomers after oral dosing and their clinical relevance. PLoS One. 2020;15(2):e0229585. doi: 10.1371/journal.pone.0229585 32108176 PMC7046201

[28] BirnbauM SM, Levintow L, KingsleY RB, Greenstein JP. Specificity of amino acid acylases. J Biol Chem. 1952;194(1):455–70. doi: 10.1016/s0021-9258(18)55898-1 14927637

[29] Lu F, Liang Q, Abi-Mosleh L, Das A, De Brabander JK, Goldstein JL, et al. Identification of NPC1 as the target of U18666A, an inhibitor of lysosomal cholesterol export and Ebola infection. Elife. 2015;4:e12177. doi: 10.7554/eLife.12177 26646182 PMC4718804

[30] Vega-Rubin-de-Celis S, Peña-Llopis S, Konda M, Brugarolas J. Multistep regulation of TFEB by MTORC1. Autophagy. 2017;13(3):464–72. doi: 10.1080/15548627.2016.1271514 28055300 PMC5361595

[31] Palmieri M, Impey S, Kang H, di Ronza A, Pelz C, Sardiello M, et al. Characterization of the CLEAR network reveals an integrated control of cellular clearance pathways. Hum Mol Genet. 2011;20(19):3852–66. doi: 10.1093/hmg/ddr306 21752829

[32] Nah J, Sung E-A, Zhai P, Zablocki D, Sadoshima J. Tfeb-mediated transcriptional regulation of autophagy induces autosis during ischemia/reperfusion in the heart. Cells. 2022;11(2):258. doi: 10.3390/cells11020258 35053374 PMC8773671

[33] Ono K, Kim SO, Han J. Susceptibility of lysosomes to rupture is a determinant for plasma membrane disruption in tumor necrosis factor alpha-induced cell death. Mol Cell Biol. 2003;23(2):665–76. doi: 10.1128/MCB.23.2.665-676.2003 12509464 PMC151543

[34] Oberle C, Huai J, Reinheckel T, Tacke M, Rassner M, Ekert PG, et al. Lysosomal membrane permeabilization and cathepsin release is a Bax/Bak-dependent, amplifying event of apoptosis in fibroblasts and monocytes. Cell Death Differ. 2010;17(7):1167–78. doi: 10.1038/cdd.2009.214 20094062

[35] Li A, Gao M, Liu B, Qin Y, Chen L, Liu H, et al. Mitochondrial autophagy: Molecular mechanisms and implications for cardiovascular disease. Cell Death Dis. 2022;13(5):444. doi: 10.1038/s41419-022-04906-6 35534453 PMC9085840

[36] Zheng HJ, Zhang X, Guo J, Zhang W, Ai S, Zhang F, et al. Lysosomal dysfunction-induced autophagic stress in diabetic kidney disease. J Cell Mol Med. 2020;24(15):8276–90. doi: 10.1111/jcmm.15301 32583573 PMC7412686

[37] Peña KA, Kiselyov K. Transition metals activate TFEB in overexpressing cells. Biochem J. 2015;470(1):65–76. doi: 10.1042/BJ20140645 26251447 PMC4613523

[38] Jiao J, Liu W, Gao G, Yang H. Serine-129 phosphorylated α-synuclein drives mitochondrial dysfunction and calcium dysregulation in Parkinson’s disease model. Front Aging Neurosci. 2025;17:1538166. doi: 10.3389/fnagi.2025.1538166 40230488 PMC11994663

[39] Song P, Chen C, Franchini R, Duong B, Wang Y-Z, Coukos R, et al. N-acetyl-l-leucine lowers α-synuclein levels and improves synaptic function in Parkinson’s disease models. J Clin Invest. 2026;136(5):e196137. doi: 10.1172/JCI196137 41766663 PMC12948429

[40] Shammas H, Kloster Fog C, Klein P, Koustrup A, Pedersen MT, Bie AS, et al. Mechanistic insights into arimoclomol mediated effects on lysosomal function in Niemann-pick type C disease. Mol Genet Metab. 2025;145(1):109103. doi: 10.1016/j.ymgme.2025.109103 40215728

