# Supplementary material for: N-acetyl-L-leucine normalizes Transcription Factor EB activity by stereospecific bidirectional modulation in a HeLa cell model of Niemann-Pick disease type C
Source: PLoS One. 2026 Jul 17;21(7):e0353834. doi: 10.1371/journal.pone.0353834 (PMC13378962; doi:10.1371/journal.pone.0353834)
Supplement: S1 Fig — These were determined by RT-qPCR. Expression was normalised to β-actin, on a scale whereβ-actin expression equals 10,000 arbitrary units. Data are shown as the mean (bars) of two biological replicates (symbols) on a logarithmic scale. MCT3 expression was not detected. (DOCX) [file pone.0353834.s001.docx]

**FIGURE S1**

**Fig. S1 Expression patterns of monocarboxylate transporters (MCT1, MCT2, MCT3, MCT4) and the aminoacylase 1 enzyme (ACY1).** These were determined by RT-qPCR. Expression was normalised to β-actin, on a scale where β-actin expression equals 10,000 arbitrary units. Data are shown as the mean (bars) of two biological replicates (symbols) on a logarithmic scale. MCT3 expression was not detected.
