# Supplementary material for: N-acetyl-L-leucine normalizes Transcription Factor EB activity by stereospecific bidirectional modulation in a HeLa cell model of Niemann-Pick disease type C
Source: PLoS One. 2026 Jul 17;21(7):e0353834. doi: 10.1371/journal.pone.0353834 (PMC13378962; doi:10.1371/journal.pone.0353834)
Supplement: S2 Fig — An NPC1 KO HeLa cell line (exon 2 NPC1-KO) was generated by the CRISPR-Cas9 technique and verified, as previously described [23]. Cell lysates were probed with primary polyclonal anti-NPC1 antibody, secondary antibodies conjugated to HRP and developed with ELC substrate. (DOCX) [file pone.0353834.s002.docx]

**FIGURE S2**

**
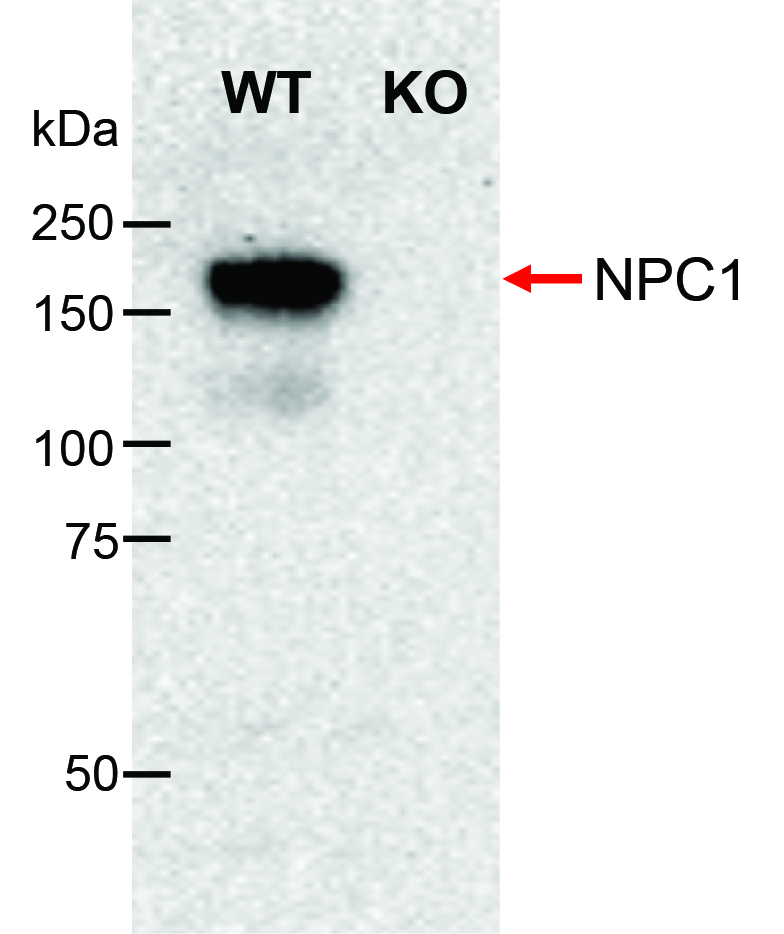
**

**Fig. S2** **Western Blot of NPC1 protein in wild-type and NPC1^-/-^ cells**

An NPC1 KO HeLa cell line (ex2 NPC1-KO) was generated by the CRISPR-cas9 technique and verified, as previously described.[23] Cell lysates were probed with primary polyclonal anti-NPC1 antibody, secondary antibodies conjugated to HRP and developed with ELC substrate.
