# Supplementary material for: N-acetyl-L-leucine normalizes Transcription Factor EB activity by stereospecific bidirectional modulation in a HeLa cell model of Niemann-Pick disease type C
Source: PLoS One. 2026 Jul 17;21(7):e0353834. doi: 10.1371/journal.pone.0353834 (PMC13378962; doi:10.1371/journal.pone.0353834)
Supplement: Attachment — Submitted filename: Response to Reviewers.docx [file pone.0353834.s004.docx]

Prof Antony Galione

Department of Pharmacology

University of Oxford

Mansfield Road

Oxford, OX1 3QT, UK

[antony.galione@pharm.ox.ac.uk](mailto:antony.galione@pharm.ox.ac.uk)

David Chau

Academic Editor

PLOS One

2 June 2026

Dear Editor,

**PONE-D-26-14007**

**N-acetyl-L-leucine normalizes Transcription Factor EB activity by stereospecific bidirectional modulation in a Hela cell model of Niemann-Pick disease type C**

Thank you for considering our manuscript. We thank all three reviewers for their helpful remarks which have improved the manuscript. We shall deal with each reviewer in turn below.

**Points raised by the Academic Editor:**

1. The manuscript has been reformatted to meet PLOS One’s style requirements.
2. Role funders took in the study: these are now fuller and clearer see (3)
3. Funding Statement: this has been expanded as has competing interest statements and author contributions/

**Funder’s role**

Support for this work was provided by charitable foundations Legacy Foundation and Fridolin Foundation and by IntraBio Inc. IntraBio Inc paid salaries for MF and TF and consultancy fees for AG, FP and GC. IntraBio Inc, Legacy Foundation and Fridolin Foundation contributed to research grants to support LLC. The funders did not have any additional role in the study design, data collection and analysis, decision to publish, or preparation of the manuscript. The specific roles of these authors are articulated in the ‘author contributions’ section.

This now has full detail

**Competing interest statements:**

**MF** and **TF** are employees and shareholders of IntraBio Inc and hold patents related to the use of acetylleucine. **MS** is Joint Chief Editor of the Journal of Neurology, Editor in Chief of Frontiers of Neuro-otology and Section Editor of F1000. He has received speaker’s honoraria from Abbott, Auris Medical, Biogen, Eisai, Grünenthal, GSK, Henning Pharma, Interacoustics, J&J, MSD, NeuroUpdate, Otometrics, Pierre-Fabre, TEVA, UCB, and Viatris. He received support for clinical studies from Decibel, U.S.A., Cure within Reach, U.S.A. and Heel, Germany. He distributes “M-glasses” and “Positional vertigo App”. He acts as a consultant for Abbott, AurisMedical, Bulbitec, Heel, Sensorion, Vifor and Vertify. He is a scientific founder, investor and shareholder of IntraBio. **MP** is an employee and shareholder of IntraBio Inc. He has received scientific advisory board honorarium from Zevra, served as journal editor for SSIEM; Journal of Inherited Metabolic Disease and JIMD Reports and Sage; Journal of Child Neurology, received publishing royalties from Wolters Kluwer, Up-To-Date, Pediatric Neurology and served as a consultant for IntraBio. He received research support from Orphazyme/Zevra, Glycomine, Azafaros, National Institute of Diabetes and Digestive and Kidney Diseases, and Idorsia Pharmaceuticals Ltd. Other authors have no relevant disclosures to report.  **AG**, **FP** and **GC** are consultants to and shareholders in IntraBio Inc.

**Author contributions:**

MF, TF: conceptualization, writing (review and editing). MF, TF: conceptualization, methodology, project administration, supervision, writing (original draft). LCD, RB, DS: investigation, methodology, writing (reviewing and editing). MF, FP, MS, WA, AG, GCC: supervision, writing (reviewing and editing). All authors had full access to all the data in the study and had final responsibility for the decision to submit for publication.

4. Submission updated to use the PLOS LaTeX template.
5. Table 1 is included on page 10.
6. Captions for Supporting Information files are included at the end of the manuscript.
7. All western blot images show the original uncropped, unadjusted image.
8. Blot image data are in Supporting Information, Fig. S2.
9. No recommendations given.

**Points raised by the Reviewers:**

**Reviewer #1**

I have one minor comment. At the end of Results section, authors describe the effects of NALL on the ratio of phosphor S122 TFEB/total TFEB in NPC1-/- cells (Figure 6B). Authors mentioned “However, the major effect was a larger significant decrease in this ratio in NPC1-/- cells, indicating that a primary effect of the drug is the inhibition of TFEB activation in NPC1-/- cells”. “Decrease in this ratio” should be changed to “increase in this ratio” to reflect the inhibition of TFEB activation.

We apologise for the confusion here and for an error: we have re-checked the statistics and there was an error in labelling the figure. We have amended this (now Fig 5C) and have re-worded this section (highlighted on page 16).

“Similarly, probing the status of the Ser122 phosphorylation site on endogenous TFEB in NPC1-/- cells, showed a significant reduction in Ser122 phosphorylation, indicating activation of TFEB (i.e. a more nuclear localisation) in the NPC1 disease cells compared to wildtype cells (Fig 5C). Upon treatment with levacetylleucine (2 mM) there was an increase in Ser122 phosphorylation in NPC1-/- cells (Fig 5C) which correlates with TFEB translocation to the cytosol upon levacetylleucine-treatment (Fig 5A, B).”

**Reviewer #2**

In Figures 2, 3, 5, and 6, the authors present quantifications of immunofluorescence data, but representative images of the cells are not shown. These images are essential for evaluating the quality and interpretation of the data. The authors are requested to include representative immunofluorescence images in the corresponding figures.

We thank the reviewer for the suggestion. We include representative single-cell images in all figures.

1. The graphs should be presented in a consistent format to facilitate interpretation of the results. Currently, Figures 5 and 6 display data in different formats, which makes comparison difficult. Please standardize the graphical representation across figures.

We have amended graphs so that they are more standardized across all figures.

2. In Figure 6, treatment with N-acetyl-leucine does not induce translocation of endogenous TFEB, in contrast to previous experiments where translocation of overexpressed TFEB was observed.
How can this discrepancy be explained?
What is the biological relevance of this finding, given that the endogenous protein is not affected?
Additionally, the authors are encouraged to analyze the effects of the enantiomers of N-acetyl-leucine on endogenous TFEB.

We have now included a suggestion for this discrepancy (highlighted on page 15). It reads:

“Whilst levacetylleucine induces a clear and pronounced translocation of overexpressed TFEB to the nucleus (Figs 1-4), it does not significantly induce translocation of endogenous TFEB. This discrepancy may be due to the difference in quantification methods comparing endogenous TFEB by antibody labelling with TFEB-EGFP (or mScarlet3) overexpression which provided a wider dynamic range which makes even subtle translocation of TFEB appear stronger. The higher TFEB-EGFP abundance amplifies the visible response and this can make NALL appear more potent in the overexpression system. Furthermore, since endogenous TFEB is often expressed at relatively low levels it may be difficult to distinguish small localization changes.”

We thank for the reviewer for the suggestion and include new data in Fig 5A and 5B analyzing the effects of the enantiomers of N-acetyl-leucine on endogenous TFEB (highlighted section on page 16).

3. In figure 6b the data appear to indicate that untreated NPC1 KO cells have lower levels of phosphorylated TFEB, while treatment restores these levels to those observed in wild-type cells. This observation should be clarified and discussed in more detail.

We have altered our discussion on page 16 (highlighted text) and hope that this has made Fig. 6B clearer.

“Similarly, probing the status of the Ser122 phosphorylation site on endogenous TFEB in NPC1-/- cells, showed a significant reduction in Ser122 phosphorylation, indicating activation of TFEB (i.e. a more nuclear localisation) in the NPC1 disease cells compared to wildtype cells (Fig 5C). Upon treatment with levacetylleucine (2 mM) there was an increase in Ser122 phosphorylation in NPC1-/- cells (Fig 5C) which correlates with TFEB translocation to the cytosol upon levacetylleucine-treatment (Fig 5A, B).”

4. To support the claim that the compound affects TFEB activity in NPC1 KO cells, the authors should assess the expression of TFEB target genes (e.g., LAMP1, cathepsins). In addition, lysosomal modulation should be evaluated, for example LAMP1 staining o lysotracker evaluation with flow cytometry analysis. These data are necessary and should be included.

We thank the reviewer for this request and we provide new data in Fig 5D, where we have assessed expression of endogenous LAMP1 upon N-acetyl-L-leucine treatment in both U-drug administered HeLa cells and in NPC1 KO HeLa cells using In-Cell Western analysis of 9 replicates for each condition (page 16). Note Fig 5D now replaces original Fig 4.

**Reviewer #3**

• Including example images and blots throughout would be nice.

We have now included representative single-cell images in all figures.

• The authors suggest that L-leucine is able to regulate TFEB via mTORC1 but no effect on mTORC1 is shown and a lack of effect of levacetylleucine on mTOR phosphorylation was previously reported (PMID: 33738443). It would be interesting to see if changes in TFEB activity correlate with changes in mTORC1 phosphorylation.

We thank the reviewer for this suggestion. We have used phosphorylation of S6K at Thr389 (assessed by In-Cell Western) as a surrogate marker for mTORC1 activation because it is one of the best-characterized direct substrates of mTORC1. Upon levacetylleucine-treatment in wild-type HeLa cells there is an increase in phosho-S6K, which strongly suggests increased mTORC1 signalling. Levacetylleucine also induced phosphorylation of mTOR at Ser2448, which reflects a positive feedback on the mTORC1 pathway because activated S6K can phosphorylate mTOR at Ser2448. Together, the observed increased phospho-mTOR (Ser2448) and increased phospho-S6K, strongly support increased mTORC1 signalling upon levaceylleucine-treatment (see Fig below).

Whilst this appears to be a clear result in wild-type cells, we have not yet extended this study into U-drug administered cells or in the NPC1 KO cells and without these further experiments we feel the data is too preliminary for a clear conclusion. We argue that this might be beyond the scope of this current manuscript and would serve well as a follow-up study with more careful controls and with additional targets of mTORC1 evaluated.

• The apparent contradiction between the reported mTORC1 hyperactivation in NPC (PMID: 28336668) and the increased TFEB activity shown here should be discussed in more detail – how do the authors reconcile this? mTORC1-independent mechanisms of TFEB regulation should also be considered/discussed

We thank the reviewer for highlighting mTORC1-independent mechanisms as well. We have included the paragraph below in our discussion (highlighted text, page 19).

“Although TFEB is best known as a target of mTORC1, there are other mTORC1-independent mechanisms that regulate its localization and activity, of which levacetylleucine may affect. These include dephosphorylation of TFEB by calcineurin, or the phosphorylation of TFEB directly without involving mTORC1 by ERK2 or GSK3β or AKT. Whilst AMPK signalling can activate TFEB both dependently and independently of mTORC1 (Zhu et al 2021).”

We hope that our amendments and new data satisfy PLOS One and the reviewers and make it now suitable for publication.

Thank you in advance.

Yours sincerely,

Prof Antony Galione
