# Supplementary material for: N-acetyl-L-leucine normalizes Transcription Factor EB activity by stereospecific bidirectional modulation in a HeLa cell model of Niemann-Pick disease type C
Source: PLoS One. 2026 Jul 17;21(7):e0353834. doi: 10.1371/journal.pone.0353834 (PMC13378962; doi:10.1371/journal.pone.0353834)
Supplement: Attachment — Submitted filename: Response to Reviewers R1.docx [file pone.0353834.s005.docx]

**PONE-D-26-14007R1**

**N-acetyl-L-leucine normalizes Transcription Factor EB activity by stereospecific bidirectional modulation in a Hela cell model of Niemann-Pick disease type C**

Thank you for considering the revision of our manuscript. We are pleased that two reviewers are happy with our modifications, and have now addressed the minor point of the third reviewer. The modified text and Figure legends are indicated in yellow on the tracked changes version of the revised manuscript.

**Points raised by the Reviewer 3**

**Reviewer #1**

As a very minor additional suggestion I think it should be made clear in the title/legend for the NALL mechanism scheme (Figure 6) that this refers to wild-type cells (and does not apply to NPC) as it shows NALL stimulating TFEB activation, whereas the authors have shown the opposite in models of NPC1 deficiency.

As the reviewer has asked for ‘WT cells’ in the legend title for Fig 6, we have now changed it and made a brief comment about Npc1^-/-^ cells in the Fig. 6 legend. Changes are marked on the yellow background.

The legend now reads: “**Fig 6. Scheme for the mechanisms of action of levacetylleucine (NALL) to enhance lysosomal and mitochondrial function in WT cells**

**Step 1** Levacetylleucine (NALL) is transported into cells via the MCT transporter family. **Step 2** Levacetylleucine inhibits the activity of the mTORC complex and reduces TFEB phosphorylation. **Step 3** Inhibition of mTOR relives its inhibition on Ca^2+^ release channels expressed in lysosomes leading to Ca^2+^ release by TPC2 and/or TRPML1 (**Step 4**). **Step 5** Lysosomal Ca^2+^ release activates calcineurin (CN) to dephosphorylate TFEB and remove its ability to bind 14-3-3 proteins allowing it to translocate to the nucleus (**Step 6**). **Step 7** TFEB bind to CLEAR gene promoters and activates lysosomal and autophagic gene expression. **Step 8** This increases lysosomal biogenesis, autophagy and lysosomal exocytosis leading to cellular clearance of stored lysosomal material (**Step 9**).

**Step 10** Levacetylleucine is metabolised to L-leucine that reactivates mTOR resulting in TFEB inactivation through phosphorylation. **Step 11** L-leucine enters metabolic pathways, where it increases mitochondrial ATP production (**Step 12**).

In NPC1^-/-^ cells, levacetylleucine has the opposite effect in that it now reduces the activity of TFEB that is constitutively enhanced in this model of NPC possibly due to the reactivation of mTORC1 by L-leucine.”

We hope that our amendments and new data satisfy PLOS One and the reviewers and make it now suitable for publication.

Thank you in advance.

Sincerely,

Prof Antony Galione
