# Supplementary material for: N-acetyl-L-leucine normalizes Transcription Factor EB activity by stereospecific bidirectional modulation in a HeLa cell model of Niemann-Pick disease type C
Source: PLoS One. 2026 Jul 17;21(7):e0353834. doi: 10.1371/journal.pone.0353834 (PMC13378962; doi:10.1371/journal.pone.0353834)
Supplement: Peer review history

# Peer review: Decision Letter 0

**DOI:** 10.1371/journal.pone.0353834.r001

**Authors:** David Chau

19 Apr 2026

PONE-D-26-14007

N-acetyl-L-leucine normalizes Transcription Factor EB activity by stereospecific bidirectional modulation in a Hela cell model of Niemann-Pick disease type C

PLOS One

Dear Dr. Galione,

Thank you for submitting your manuscript to PLOS ONE. After careful consideration, we feel that it has merit but does not fully meet PLOS ONE’s publication criteria as it currently stands. Therefore, we invite you to submit a revised version of the manuscript that addresses the points raised during the review process.

Please submit your revised manuscript by Jun 03 2026 11:59PM. If you will need more time than this to complete your revisions, please reply to this message or contact the journal office at plosone@plos.org. When you're ready to submit your revision, log on to https://www.editorialmanager.com/pone/ and select the 'Submissions Needing Revision' folder to locate your manuscript file.

Please include the following items when submitting your revised manuscript:

- A letter that responds to each point raised by the academic editor and reviewer(s). You should upload this letter as a separate file labeled 'Response to Reviewers'.
- A marked-up copy of your manuscript that highlights changes made to the original version. You should upload this as a separate file labeled 'Revised Manuscript with Track Changes'.
- An unmarked version of your revised paper without tracked changes. You should upload this as a separate file labeled 'Manuscript'.

If you would like to make changes to your financial disclosure, please include your updated statement in your cover letter. Guidelines for resubmitting your figure files are available below the reviewer comments at the end of this letter.

If applicable, we recommend that you deposit your laboratory protocols in protocols.io to enhance the reproducibility of your results. Protocols.io assigns your protocol its own identifier (DOI) so that it can be cited independently in the future. For instructions see: https://journals.plos.org/plosone/s/submission-guidelines#loc-laboratory-protocols. Additionally, PLOS ONE offers an option for publishing peer-reviewed Lab Protocol articles, which describe protocols hosted on protocols.io. Read more information on sharing protocols at https://plos.org/protocols?utm_medium=editorial-email&utm_source=authorletters&utm_campaign=protocols.

As the corresponding author, your ORCID iD is verified in the submission system and will appear in the published article. PLOS supports the use of ORCID, and we encourage all coauthors to register for an ORCID iD and use it as well. Please encourage your coauthors to verify their ORCID iD within the submission system before final acceptance, as unverified ORCID iDs will not appear in the published article. *Only*  the individual author can complete the verification step; PLOS staff *cannot*  verify ORCID iDs on behalf of authors.

We look forward to receiving your revised manuscript.

Kind regards,

David Chau

Academic Editor

PLOS One

Journal Requirements:

When submitting your revision, we need you to address these additional requirements.

1. Please ensure that your manuscript meets PLOS ONE's style requirements, including those for file naming. The PLOS ONE style templates can be found at

https://journals.plos.org/plosone/s/file?id=wjVg/PLOSOne_formatting_sample_main_body.pdf and

https://journals.plos.org/plosone/s/file?id=ba62/PLOSOne_formatting_sample_title_authors_affiliations.pdf

2. Thank you for stating the following financial disclosure: [Fridolin Foundation, Legacy Foundation].

Please state what role the funders took in the study.  If the funders had no role, please state: ""The funders had no role in study design, data collection and analysis, decision to publish, or preparation of the manuscript.""

If this statement is not correct you must amend it as needed.

Please include this amended Role of Funder statement in your cover letter; we will change the online submission form on your behalf.

3. Thank you for stating the following in the Competing Interests section: [I have read the journal's policy and the authors of this manuscript have the following competing interests: Grant Churchill, Antony Galione and Frances Platt are academic co-founders and consultants of IntraBio Inc. Mallory Factor, Taylor Fields and Marc Patterson are employees of IntraBio Inc. All the employees and consultants listed above with Michael Strupp are shareholders of IntraBio, Inc.].

We note that one or more of the authors are employed by a commercial company: [IntraBio Inc]

1. Please provide an amended Funding Statement declaring this commercial affiliation, as well as a statement regarding the Role of Funders in your study. If the funding organization did not play a role in the study design, data collection and analysis, decision to publish, or preparation of the manuscript and only provided financial support in the form of authors' salaries and/or research materials, please review your statements relating to the author contributions, and ensure you have specifically and accurately indicated the role(s) that these authors had in your study. You can update author roles in the Author Contributions section of the online submission form.

Please also include the following statement within your amended Funding Statement.

“The funder provided support in the form of salaries for authors [insert relevant initials], but did not have any additional role in the study design, data collection and analysis, decision to publish, or preparation of the manuscript. The specific roles of these authors are articulated in the ‘author contributions’ section.”

If your commercial affiliation did play a role in your study, please state and explain this role within your updated Funding Statement.

2. Please also provide an updated Competing Interests Statement declaring this commercial affiliation along with any other relevant declarations relating to employment, consultancy, patents, products in development, or marketed products, etc.

Within your Competing Interests Statement, please confirm that this commercial affiliation does not alter your adherence to all PLOS ONE policies on sharing data and materials by including the following statement: ""This does not alter our adherence to  PLOS ONE policies on sharing data and materials.” (as detailed online in our guide for authors http://journals.plos.org/plosone/s/competing-interests) . If this adherence statement is not accurate and  there are restrictions on sharing of data and/or materials, please state these. Please note that we cannot proceed with consideration of your article until this information has been declared.

Please include both an updated Funding Statement and Competing Interests Statement in your cover letter. We will change the online submission form on your behalf.

4. Please update your submission to use the PLOS LaTeX template. The template and more information on our requirements for LaTeX submissions can be found at http://journals.plos.org/plosone/s/latex.

5. Please include a copy of Table 1 which you refer to in your text on page 10.

6. Please include captions for your Supporting Information files at the end of your manuscript, and update any in-text citations to match accordingly. Please see our Supporting Information guidelines for more information: http://journals.plos.org/plosone/s/supporting-information.

7. PLOS ONE now requires that authors provide the original uncropped and unadjusted images underlying all blot or gel results reported in a submission’s figures or Supporting Information files. This policy and the journal’s other requirements for blot/gel reporting and figure preparation are described in detail at https://journals.plos.org/plosone/s/figures#loc-blot-and-gel-reporting-requirements and https://journals.plos.org/plosone/s/figures#loc-preparing-figures-from-image-files. When you submit your revised manuscript, please ensure that your figures adhere fully to these guidelines and provide the original underlying images for all blot or gel data reported in your submission. See the following link for instructions on providing the original image data: https://journals.plos.org/plosone/s/figures#loc-original-images-for-blots-and-gels.

8. In your cover letter, please note whether your blot/gel image data are in Supporting Information or posted at a public data repository, provide the repository URL if relevant, and provide specific details as to which raw blot/gel images, if any, are not available. Email us at plosone@plos.org if you have any questions.

9. If the reviewer comments include a recommendation to cite specific previously published works, please review and evaluate these publications to determine whether they are relevant and should be cited. There is no requirement to cite these works unless the editor has indicated otherwise.

[Note: HTML markup is below. Please do not edit.]

Reviewers' comments:

Reviewer's Responses to Questions

**Comments to the Author**

1. Is the manuscript technically sound, and do the data support the conclusions?

The manuscript must describe a technically sound piece of scientific research with data that supports the conclusions. Experiments must have been conducted rigorously, with appropriate controls, replication, and sample sizes. The conclusions must be drawn appropriately based on the data presented.

Reviewer #1: Yes

Reviewer #2: Partly

Reviewer #3: Yes

**********

2. Has the statistical analysis been performed appropriately and rigorously?

Reviewer #1: Yes

Reviewer #2: Yes

Reviewer #3: Yes

**********

3. Have the authors made all data underlying the findings in their manuscript fully available?

The PLOS Data policy requires authors to make all data underlying the findings described in their manuscript fully available without restriction, with rare exception (please refer to the Data Availability Statement in the manuscript PDF file). The data should be provided as part of the manuscript or its supporting information, or deposited to a public repository. For example, in addition to summary statistics, the data points behind means, medians and variance measures should be available. If there are restrictions on publicly sharing data—e.g. participant privacy or use of data from a third party—those must be specified.

Reviewer #1: Yes

Reviewer #2: Yes

Reviewer #3: Yes

**********

4. Is the manuscript presented in an intelligible fashion and written in standard English?

PLOS ONE does not copyedit accepted manuscripts, so the language in submitted articles must be clear, correct, and unambiguous. Any typographical or grammatical errors should be corrected at revision, so please note any specific errors here.

Reviewer #1: Yes

Reviewer #2: Yes

Reviewer #3: Yes

**********

5. Review Comments to the Author

Please use the space provided to explain your answers to the questions above. You may also include additional comments for the author, including concerns about dual publication, research ethics, or publication ethics. (Please upload your review as an attachment if it exceeds 20,000 characters)

Reviewer #1: This is a very interesting work showing a potential for N-acetyl-L-leucine for NPC disease as well as other neurodegenerative diseases. The manuscript is clear and well written.

I have one minor comment. At the end of Results section, authors describe the effects of NALL on the ratio of phosphor S122 TFEB/total TFEB in NPC1-/- cells (Figure 6B). Authors mentioned “However, the major effect was a larger significant decrease in this ratio in NPC1-/- cells, indicating that a primary effect of the drug is the inhibition of TFEB activation in NPC1-/- cells”. “Decrease in this ratio” should be changed to “increase in this ratio” to reflect the inhibition of TFEB activation.

Reviewer #2: In this manuscript, Davis et al. investigate the modulation of TFEB by N-acetyl-leucine in wild-type cells and models of Niemann-Pick disease type C. While the study addresses a relevant topic for the disease, additional experimental evidence and clarification of key points are required to strengthen the manuscript.

In Figures 2, 3, 5, and 6, the authors present quantifications of immunofluorescence data, but representative images of the cells are not shown. These images are essential for evaluating the quality and interpretation of the data. The authors are requested to include representative immunofluorescence images in the corresponding figures.

1. The graphs should be presented in a consistent format to facilitate interpretation of the results. Currently, Figures 5 and 6 display data in different formats, which makes comparison difficult. Please standardize the graphical representation across figures.

2. In Figure 6, treatment with N-acetyl-leucine does not induce translocation of endogenous TFEB, in contrast to previous experiments where translocation of overexpressed TFEB was observed.

How can this discrepancy be explained?

What is the biological relevance of this finding, given that the endogenous protein is not affected?

Additionally, the authors are encouraged to analyze the effects of the enantiomers of N-acetyl-leucine on endogenous TFEB.

3. In figure 6b the data appear to indicate that untreated NPC1 KO cells have lower levels of phosphorylated TFEB, while treatment restores these levels to those observed in wild-type cells. This observation should be clarified and discussed in more detail.

4. To support the claim that the compound affects TFEB activity in NPC1 KO cells, the authors should assess the expression of TFEB target genes (e.g., LAMP1, cathepsins). In addition, lysosomal modulation should be evaluated, for example LAMP1 staining o lysotracker evaluation with flow cytometry analysis. These data are necessary and should be included.

Reviewer #3: Levacetylleucine (N-Acetyl-L-leucine) is an approved therapeutic for the lysosomal storage disease Niemann-Pick disease type C (NPC) and restores both lysosome and mitochondrial function in NPC models, though the precise mechanism of action remains unclear.

Here the authors build on their previous important finding that N-acetylation increases leucine uptake via monocarboxylate transporters, to show that levacetylleucine normalises aberrant activity of TFEB, the master regulator of lysosome biogenesis and autophagy. They find that treatment with levacetylleucine rapidly activates overexpressed, but not endogenous, TFEB in control cells. Since TFEB activation has been proposed as a potential therapeutic approach for NPC, taken alone this could implicate TFEB activation in the mechanism of action of levacetylleucine. However, increased TFEB activity has been reported in NPC which the authors here confirm in their models. Importantly they go on to show that rather than further increasing the already hyperactive TFEB, levacetylleucine treatment reduces TFEB activity, normalising it almost to control levels, consistent with restoration of lysosomal homeostasis in levacetylleucine-treated models of NPC. Interestingly effects on TFEB are specific to levacetylleucine, with the D-enantiomer or NADLL racemate having no effect. Overall this is a significant finding that will be of great interest to the community.

The manuscript is very well-written and presents robust data with rigorous experimental design, including validation of findings by multiple methods, giving a high level of confidence in the findings.

Minor comments/suggestions:

• Including example images and blots throughout would be nice.

• The authors suggest that L-leucine is able to regulate TFEB via mTORC1 but no effect on mTORC1 is shown and a lack of effect of levacetylleucine on mTOR phosphorylation was previously reported (PMID: 33738443). It would be interesting to see if changes in TFEB activity correlate with changes in mTORC1 phosphorylation.

• The apparent contradiction between the reported mTORC1 hyperactivation in NPC (PMID: 28336668) and the increased TFEB activity shown here should be discussed in more detail – how do the authors reconcile this? mTORC1-independent mechanisms of TFEB regulation should also be considered/discussed.

**********

6. PLOS authors have the option to publish the peer review history of their article (what does this mean?). If published, this will include your full peer review and any attached files.

If you choose “no”, your identity will remain anonymous but your review may still be made public.

**Do you want your identity to be public for this peer review?**  For information about this choice, including consent withdrawal, please see our Privacy Policy.

Reviewer #1: **Yes:**  Byeongwoon Song

Reviewer #2: No

Reviewer #3: No

**********

[NOTE: If reviewer comments were submitted as an attachment file, they will be attached to this email and accessible via the submission site. Please log into your account, locate the manuscript record, and check for the action link "View Attachments". If this link does not appear, there are no attachment files.]

To ensure your figures meet our technical requirements, please review our figure guidelines: https://journals.plos.org/plosone/s/figures

You may also use PLOS’s free figure tool, NAAS, to help you prepare publication quality figures: https://journals.plos.org/plosone/s/figures#loc-tools-for-figure-preparation.

NAAS will assess whether your figures meet our technical requirements by comparing each figure against our figure specifications.

# Author comment: Author response to Decision Letter 1

**DOI:** 10.1371/journal.pone.0353834.r002

3 Jun 2026

Points raised by the Reviewers:

Reviewer #1

I have one minor comment. At the end of Results section, authors describe the effects of NALL on the ratio of phosphor S122 TFEB/total TFEB in NPC1-/- cells (Figure 6B). Authors mentioned “However, the major effect was a larger significant decrease in this ratio in NPC1-/- cells, indicating that a primary effect of the drug is the inhibition of TFEB activation in NPC1-/- cells”. “Decrease in this ratio” should be changed to “increase in this ratio” to reflect the inhibition of TFEB activation.

We apologise for the confusion here and for an error: we have re-checked the statistics and there was an error in labelling the figure. We have amended this (now Fig 5C) and have re-worded this section (highlighted on page 16).

“Similarly, probing the status of the Ser122 phosphorylation site on endogenous TFEB in NPC1-/- cells, showed a significant reduction in Ser122 phosphorylation, indicating activation of TFEB (i.e. a more nuclear localisation) in the NPC1 disease cells compared to wildtype cells (Fig 5C). Upon treatment with levacetylleucine (2 mM) there was an increase in Ser122 phosphorylation in NPC1-/- cells (Fig 5C) which correlates with TFEB translocation to the cytosol upon levacetylleucine-treatment (Fig 5A, B).”

Reviewer #2

In Figures 2, 3, 5, and 6, the authors present quantifications of immunofluorescence data, but representative images of the cells are not shown. These images are essential for evaluating the quality and interpretation of the data. The authors are requested to include representative immunofluorescence images in the corresponding figures.

We thank the reviewer for the suggestion. We include representative single-cell images in all figures.

1. The graphs should be presented in a consistent format to facilitate interpretation of the results. Currently, Figures 5 and 6 display data in different formats, which makes comparison difficult. Please standardize the graphical representation across figures.

We have amended graphs so that they are more standardized across all figures.

2. In Figure 6, treatment with N-acetyl-leucine does not induce translocation of endogenous TFEB, in contrast to previous experiments where translocation of overexpressed TFEB was observed.

How can this discrepancy be explained?

What is the biological relevance of this finding, given that the endogenous protein is not affected?

Additionally, the authors are encouraged to analyze the effects of the enantiomers of N-acetyl-leucine on endogenous TFEB.

We have now included a suggestion for this discrepancy (highlighted on page 15). It reads:

“Whilst levacetylleucine induces a clear and pronounced translocation of overexpressed TFEB to the nucleus (Figs 1-4), it does not significantly induce translocation of endogenous TFEB. This discrepancy may be due to the difference in quantification methods comparing endogenous TFEB by antibody labelling with TFEB-EGFP (or mScarlet3) overexpression which provided a wider dynamic range which makes even subtle translocation of TFEB appear stronger. The higher TFEB-EGFP abundance amplifies the visible response and this can make NALL appear more potent in the overexpression system. Furthermore, since endogenous TFEB is often expressed at relatively low levels it may be difficult to distinguish small localization changes.”

We thank for the reviewer for the suggestion and include new data in Fig 5A and 5B analyzing the effects of the enantiomers of N-acetyl-leucine on endogenous TFEB (highlighted section on page 16).

3. In figure 6b the data appear to indicate that untreated NPC1 KO cells have lower levels of phosphorylated TFEB, while treatment restores these levels to those observed in wild-type cells. This observation should be clarified and discussed in more detail.

We have altered our discussion on page 16 (highlighted text) and hope that this has made Fig. 6B clearer.

“Similarly, probing the status of the Ser122 phosphorylation site on endogenous TFEB in NPC1-/- cells, showed a significant reduction in Ser122 phosphorylation, indicating activation of TFEB (i.e. a more nuclear localisation) in the NPC1 disease cells compared to wildtype cells (Fig 5C). Upon treatment with levacetylleucine (2 mM) there was an increase in Ser122 phosphorylation in NPC1-/- cells (Fig 5C) which correlates with TFEB translocation to the cytosol upon levacetylleucine-treatment (Fig 5A, B).”

4. To support the claim that the compound affects TFEB activity in NPC1 KO cells, the authors should assess the expression of TFEB target genes (e.g., LAMP1, cathepsins). In addition, lysosomal modulation should be evaluated, for example LAMP1 staining o lysotracker evaluation with flow cytometry analysis. These data are necessary and should be included.

We thank the reviewer for this request and we provide new data in Fig 5D, where we have assessed expression of endogenous LAMP1 upon N-acetyl-L-leucine treatment in both U-drug administered HeLa cells and in NPC1 KO HeLa cells using In-Cell Western analysis of 9 replicates for each condition (page 16). Note Fig 5D now replaces original Fig 4.

Reviewer #3

• Including example images and blots throughout would be nice.

We have now included representative single-cell images in all figures.

• The authors suggest that L-leucine is able to regulate TFEB via mTORC1 but no effect on mTORC1 is shown and a lack of effect of levacetylleucine on mTOR phosphorylation was previously reported (PMID: 33738443). It would be interesting to see if changes in TFEB activity correlate with changes in mTORC1 phosphorylation.

We thank the reviewer for this suggestion. We have used phosphorylation of S6K at Thr389 (assessed by In-Cell Western) as a surrogate marker for mTORC1 activation because it is one of the best-characterized direct substrates of mTORC1. Upon levacetylleucine-treatment in wild-type HeLa cells there is an increase in phosho-S6K, which strongly suggests increased mTORC1 signalling. Levacetylleucine also induced phosphorylation of mTOR at Ser2448, which reflects a positive feedback on the mTORC1 pathway because activated S6K can phosphorylate mTOR at Ser2448. Together, the observed increased phospho-mTOR (Ser2448) and increased phospho-S6K, strongly support increased mTORC1 signalling upon levaceylleucine-treatment (see Fig below).

Whilst this appears to be a clear result in wild-type cells, we have not yet extended this study into U-drug administered cells or in the NPC1 KO cells and without these further experiments we feel the data is too preliminary for a clear conclusion. We argue that this might be beyond the scope of this current manuscript and would serve well as a follow-up study with more careful controls and with additional targets of mTORC1 evaluated.

• The apparent contradiction between the reported mTORC1 hyperactivation in NPC (PMID: 28336668) and the increased TFEB activity shown here should be discussed in more detail – how do the authors reconcile this? mTORC1-independent mechanisms of TFEB regulation should also be considered/discussed

We thank the reviewer for highlighting mTORC1-independent mechanisms as well. We have included the paragraph below in our discussion (highlighted text, page 19).

“Although TFEB is best known as a target of mTORC1, there are other mTORC1-independent mechanisms that regulate its localization and activity, of which levacetylleucine may affect. These include dephosphorylation of TFEB by calcineurin, or the phosphorylation of TFEB directly without involving mTORC1 by ERK2 or GSK3β or AKT. Whilst AMPK signalling can activate TFEB both dependently and independently of mTORC1 (Zhu et al 2021).”

Attachment Submitted filename: Response to Reviewers.docx

# Peer review: Decision Letter 1

**DOI:** 10.1371/journal.pone.0353834.r003

**Authors:** David Chau, David Chau

15 Jun 2026

PONE-D-26-14007R1

N-acetyl-L-leucine normalizes Transcription Factor EB activity by stereospecific bidirectional modulation in a Hela cell model of Niemann-Pick disease type C

PLOS One

Dear Dr. Galione,

Thank you for submitting your manuscript to PLOS ONE. After careful consideration, we feel that it has merit but does not fully meet PLOS ONE’s publication criteria as it currently stands. Therefore, we invite you to submit a revised version of the manuscript that addresses the points raised during the review process.

Please submit your revised manuscript by Jul 30 2026 11:59PM. If you will need more time than this to complete your revisions, please reply to this message or contact the journal office at plosone@plos.org. When you're ready to submit your revision, log on to https://www.editorialmanager.com/pone/ and select the 'Submissions Needing Revision' folder to locate your manuscript file.

Please include the following items when submitting your revised manuscript:

- A letter that responds to each point raised by the academic editor and reviewer(s). You should upload this letter as a separate file labeled 'Response to Reviewers'.
- A marked-up copy of your manuscript that highlights changes made to the original version. You should upload this as a separate file labeled 'Revised Manuscript with Track Changes'.
- An unmarked version of your revised paper without tracked changes. You should upload this as a separate file labeled 'Manuscript'.

If you would like to make changes to your financial disclosure, please include your updated statement in your cover letter. Guidelines for resubmitting your figure files are available below the reviewer comments at the end of this letter.

If applicable, we recommend that you deposit your laboratory protocols in protocols.io to enhance the reproducibility of your results. Protocols.io assigns your protocol its own identifier (DOI) so that it can be cited independently in the future. For instructions see: https://journals.plos.org/plosone/s/submission-guidelines#loc-laboratory-protocols. Additionally, PLOS ONE offers an option for publishing peer-reviewed Lab Protocol articles, which describe protocols hosted on protocols.io. Read more information on sharing protocols at https://plos.org/protocols?utm_medium=editorial-email&utm_source=authorletters&utm_campaign=protocols.

As the corresponding author, your ORCID iD is verified in the submission system and will appear in the published article. PLOS supports the use of ORCID, and we encourage all coauthors to register for an ORCID iD and use it as well. Please encourage your coauthors to verify their ORCID iD within the submission system before final acceptance, as unverified ORCID iDs will not appear in the published article. *Only*  the individual author can complete the verification step; PLOS staff *cannot*  verify ORCID iDs on behalf of authors.

We look forward to receiving your revised manuscript.

Kind regards,

David Chau

Academic Editor

PLOS One

Journal Requirements:

If the reviewer comments include a recommendation to cite specific previously published works, please review and evaluate these publications to determine whether they are relevant and should be cited. There is no requirement to cite these works unless the editor has indicated otherwise.

Please review your reference list to ensure that it is complete and correct. If you have cited papers that have been retracted, please include the rationale for doing so in the manuscript text, or remove these references and replace them with relevant current references. Any changes to the reference list should be mentioned in the rebuttal letter that accompanies your revised manuscript. If you need to cite a retracted article, indicate the article’s retracted status in the References list and also include a citation and full reference for the retraction notice.

[Note: HTML markup is below. Please do not edit.]

Reviewers' comments:

Reviewer's Responses to Questions

**Comments to the Author**

1. If the authors have adequately addressed your comments raised in a previous round of review and you feel that this manuscript is now acceptable for publication, you may indicate that here to bypass the “Comments to the Author” section, enter your conflict of interest statement in the “Confidential to Editor” section, and submit your "Accept" recommendation.

Reviewer #2: All comments have been addressed

Reviewer #3: (No Response)

**********

2. Is the manuscript technically sound, and do the data support the conclusions?

The manuscript must describe a technically sound piece of scientific research with data that supports the conclusions. Experiments must have been conducted rigorously, with appropriate controls, replication, and sample sizes. The conclusions must be drawn appropriately based on the data presented.

Reviewer #2: Yes

Reviewer #3: (No Response)

**********

3. Has the statistical analysis been performed appropriately and rigorously?

Reviewer #2: Yes

Reviewer #3: (No Response)

**********

4. Have the authors made all data underlying the findings in their manuscript fully available?

The PLOS Data policy requires authors to make all data underlying the findings described in their manuscript fully available without restriction, with rare exception (please refer to the Data Availability Statement in the manuscript PDF file). The data should be provided as part of the manuscript or its supporting information, or deposited to a public repository. For example, in addition to summary statistics, the data points behind means, medians and variance measures should be available. If there are restrictions on publicly sharing data—e.g. participant privacy or use of data from a third party—those must be specified.

Reviewer #2: (No Response)

Reviewer #3: (No Response)

**********

5. Is the manuscript presented in an intelligible fashion and written in standard English?

PLOS ONE does not copyedit accepted manuscripts, so the language in submitted articles must be clear, correct, and unambiguous. Any typographical or grammatical errors should be corrected at revision, so please note any specific errors here.

Reviewer #2: Yes

Reviewer #3: (No Response)

**********

6. Review Comments to the Author

Please use the space provided to explain your answers to the questions above. You may also include additional comments for the author, including concerns about dual publication, research ethics, or publication ethics. (Please upload your review as an attachment if it exceeds 20,000 characters)

Reviewer #2: (No Response)

Reviewer #3: Comments have mostly been addressed (with the exception of effects on mTORC1 in NPC) and the manuscript is improved. As a very minor additional suggestion I think it should be made clear in the title/legend for the NALL mechanism scheme (Figure 6) that this refers to wild-type cells (and does not apply to NPC) as it shows NALL stimulating TFEB activation, whereas the authors have shown the opposite in models of NPC1 deficiency.

**********

7. PLOS authors have the option to publish the peer review history of their article (what does this mean?). If published, this will include your full peer review and any attached files.

If you choose “no”, your identity will remain anonymous but your review may still be made public.

**Do you want your identity to be public for this peer review?**  For information about this choice, including consent withdrawal, please see our Privacy Policy.

Reviewer #2: No

Reviewer #3: No

**********

[NOTE: If reviewer comments were submitted as an attachment file, they will be attached to this email and accessible via the submission site. Please log into your account, locate the manuscript record, and check for the action link "View Attachments". If this link does not appear, there are no attachment files.]

To ensure your figures meet our technical requirements, please review our figure guidelines: https://journals.plos.org/plosone/s/figures

You may also use PLOS’s free figure tool, NAAS, to help you prepare publication quality figures: https://journals.plos.org/plosone/s/figures#loc-tools-for-figure-preparation.

NAAS will assess whether your figures meet our technical requirements by comparing each figure against our figure specifications.

# Author comment: Author response to Decision Letter 2

**DOI:** 10.1371/journal.pone.0353834.r004

29 Jun 2026

Thank you for considering the revision of our manuscript. We are pleased that two reviewers are happy with our modifications, and have now addressed the minor point of the third reviewer. The modified text and Figure legends are indicated in yellow on the tracked changes version of the revised manuscript.

Points raised by the Reviewer 3

Reviewer #1

As a very minor additional suggestion I think it should be made clear in the title/legend for the NALL mechanism scheme (Figure 6) that this refers to wild-type cells (and does not apply to NPC) as it shows NALL stimulating TFEB activation, whereas the authors have shown the opposite in models of NPC1 deficiency.

As the reviewer has asked for ‘WT cells’ in the legend title for Fig 6, we have now changed it and made a brief comment about Npc1-/- cells in the Fig. 6 legend. Changes are marked on the yellow background.

The legend now reads: “Fig 6. Scheme for the mechanisms of action of levacetylleucine (NALL) to enhance lysosomal and mitochondrial function in WT cells

Step 1 Levacetylleucine (NALL) is transported into cells via the MCT transporter family. Step 2 Levacetylleucine inhibits the activity of the mTORC complex and reduces TFEB phosphorylation. Step 3 Inhibition of mTOR relives its inhibition on Ca2+ release channels expressed in lysosomes leading to Ca2+ release by TPC2 and/or TRPML1 (Step 4). Step 5 Lysosomal Ca2+ release activates calcineurin (CN) to dephosphorylate TFEB and remove its ability to bind 14-3-3 proteins allowing it to translocate to the nucleus (Step 6). Step 7 TFEB bind to CLEAR gene promoters and activates lysosomal and autophagic gene expression. Step 8 This increases lysosomal biogenesis, autophagy and lysosomal exocytosis leading to cellular clearance of stored lysosomal material (Step 9).

Step 10 Levacetylleucine is metabolised to L-leucine that reactivates mTOR resulting in TFEB inactivation through phosphorylation. Step 11 L-leucine enters metabolic pathways, where it increases mitochondrial ATP production (Step 12).

In NPC1-/- cells, levacetylleucine has the opposite effect in that it now reduces the activity of TFEB that is constitutively enhanced in this model of NPC possibly due to the reactivation of mTORC1 by L-leucine.”

Attachment Submitted filename: Response to Reviewers R1.docx

# Peer review: Decision Letter 2

**DOI:** 10.1371/journal.pone.0353834.r005

**Authors:** David Chau, David Chau, David Chau

1 Jul 2026

N-acetyl-L-leucine normalizes Transcription Factor EB activity by stereospecific bidirectional modulation in a Hela cell model of Niemann-Pick disease type C

PONE-D-26-14007R2

Dear Dr. Galione,

We’re pleased to inform you that your manuscript has been judged scientifically suitable for publication and will be formally accepted for publication once it meets all outstanding technical requirements.

Within one week, you’ll receive an e-mail detailing the required amendments. When these have been addressed, you’ll receive a formal acceptance letter and your manuscript will be scheduled for publication.

An invoice will be generated when your article is formally accepted. Please note, if your institution has a publishing partnership with PLOS and your article meets the relevant criteria, all or part of your publication costs will be covered. Please make sure your user information is up-to-date by logging into Editorial Manager at Editorial Manager® and clicking the ‘Update My Information' link at the top of the page. For questions related to billing, please contact billing support.

If your institution or institutions have a press office, please notify them about your upcoming paper to help maximize its impact. If they’ll be preparing press materials, please inform our press team as soon as possible -- no later than 48 hours after receiving the formal acceptance. Your manuscript will remain under strict press embargo until 2 pm Eastern Time on the date of publication. For more information, please contact onepress@plos.org.

Kind regards,

David Chau

Academic Editor

PLOS One

Additional Editor Comments (optional):

Reviewers' comments:

Reviewer's Responses to Questions

**Comments to the Author**

1. If the authors have adequately addressed your comments raised in a previous round of review and you feel that this manuscript is now acceptable for publication, you may indicate that here to bypass the “Comments to the Author” section, enter your conflict of interest statement in the “Confidential to Editor” section, and submit your "Accept" recommendation.

Reviewer #1: (No Response)

Reviewer #3: (No Response)

**********

2. Is the manuscript technically sound, and do the data support the conclusions?

The manuscript must describe a technically sound piece of scientific research with data that supports the conclusions. Experiments must have been conducted rigorously, with appropriate controls, replication, and sample sizes. The conclusions must be drawn appropriately based on the data presented.

Reviewer #1: Yes

Reviewer #3: Yes

**********

3. Has the statistical analysis been performed appropriately and rigorously?

Reviewer #1: Yes

Reviewer #3: Yes

**********

4. Have the authors made all data underlying the findings in their manuscript fully available?

The PLOS Data policy requires authors to make all data underlying the findings described in their manuscript fully available without restriction, with rare exception (please refer to the Data Availability Statement in the manuscript PDF file). The data should be provided as part of the manuscript or its supporting information, or deposited to a public repository. For example, in addition to summary statistics, the data points behind means, medians and variance measures should be available. If there are restrictions on publicly sharing data—e.g. participant privacy or use of data from a third party—those must be specified.

Reviewer #1: Yes

Reviewer #3: Yes

**********

5. Is the manuscript presented in an intelligible fashion and written in standard English?

PLOS ONE does not copyedit accepted manuscripts, so the language in submitted articles must be clear, correct, and unambiguous. Any typographical or grammatical errors should be corrected at revision, so please note any specific errors here.

Reviewer #1: Yes

Reviewer #3: Yes

**********

6. Review Comments to the Author

Please use the space provided to explain your answers to the questions above. You may also include additional comments for the author, including concerns about dual publication, research ethics, or publication ethics. (Please upload your review as an attachment if it exceeds 20,000 characters)

Reviewer #1: (No Response)

Reviewer #3: (No Response)

**********

7. PLOS authors have the option to publish the peer review history of their article (what does this mean?). If published, this will include your full peer review and any attached files.

If you choose “no”, your identity will remain anonymous but your review may still be made public.

**Do you want your identity to be public for this peer review?**  For information about this choice, including consent withdrawal, please see our Privacy Policy.

Reviewer #1: No

Reviewer #3: No

**********

# Editor report: Acceptance letter

**DOI:** 10.1371/journal.pone.0353834.r006

**Authors:** David Chau, David Chau, David Chau

PONE-D-26-14007R2

PLOS One

Dear Dr. Galione,

I'm pleased to inform you that your manuscript has been deemed suitable for publication in PLOS One. Congratulations! Your manuscript is now being handed over to our production team.

At this stage, our production department will prepare your paper for publication. This includes ensuring the following:

* All references, tables, and figures are properly cited

* All relevant supporting information is included in the manuscript submission,

* There are no issues that prevent the paper from being properly typeset

You will receive further instructions from the production team, including instructions on how to review your proof when it is ready. Please keep in mind that we are working through a large volume of accepted articles, so please give us a few days to review your paper and let you know the next and final steps.

Lastly, if your institution or institutions have a press office, please let them know about your upcoming paper now to help maximize its impact. If they'll be preparing press materials, please inform our press team within the next 48 hours. Your manuscript will remain under strict press embargo until 2 pm Eastern Time on the date of publication. For more information, please contact onepress@plos.org.

You will receive an invoice from PLOS for your publication fee after your manuscript has reached the completed accept phase. If you receive an email requesting payment before acceptance or for any other service, this may be a phishing scheme. Learn how to identify phishing emails and protect your accounts at https://explore.plos.org/phishing.

If we can help with anything else, please email us at customercare@plos.org.

Thank you for submitting your work to PLOS ONE and supporting open access.

Kind regards,

PLOS ONE Editorial Office Staff

on behalf of

Dr. David Chau

Academic Editor

PLOS One
